# Expression, purification, and functional characterization of soluble recombinant full-length simian immunodeficiency virus (SIV) Pr55^Gag^

**DOI:** 10.1016/j.heliyon.2023.e12892

**Published:** 2023-01-10

**Authors:** Vineeta N. Pillai, Lizna Mohamed Ali, Suresha G. Prabhu, Anjana Krishnan, Saeed Tariq, Farah Mustafa, Tahir A. Rizvi

**Affiliations:** a Department of Microbiology & Immunology, College of Medicine and Health Sciences (CMHS), United Arab Emirates University, Al Ain, United Arab Emirates; b Department of Anatomy, College of Medicine and Health Sciences (CMHS), United Arab Emirates University, Al Ain, United Arab Emirates; c Department of Biochemistry, College of Medicine and Health Sciences (CMHS), United Arab Emirates University, Al Ain, United Arab Emirates; d Zayed Center for Health Sciences, United Arab Emirates University, Al Ain, United Arab Emirates

**Keywords:** Retroviruses, Simian immunodeficiency virus (SIV), Protein purification and expression, SIV Pr55^Gag^ His_6_-tagged fusion protein purification, RNA binding protein, Chromatography, *In vitro* and *in vivo* viral particle assembly, RNA packaging

## Abstract

The simian immunodeficiency virus (SIV) precursor polypeptide Pr55^Gag^ drives viral assembly and facilitates specific recognition and packaging of the SIV genomic RNA (gRNA) into viral particles. While several studies have tried to elucidate the role of SIV Pr55^Gag^ by expressing its different components independently, studies using full-length SIV Pr55^Gag^ have not been conducted, primarily due to the unavailability of purified and biologically active full-length SIV Pr55^Gag^. We successfully expressed soluble, full-length SIV Pr55^Gag^ with His_6_-tag in bacteria and purified it using affinity and gel filtration chromatography. In the process, we identified within Gag, a second in-frame start codon downstream of a putative Shine-Dalgarno-like sequence resulting in an additional truncated form of Gag. Synonymously mutating this sequence allowed expression of full-length Gag in its native form. The purified Gag assembled into virus-like particles (VLPs) *in vitro* in the presence of nucleic acids, revealing its biological functionality. *In vivo* experiments also confirmed formation of functional VLPs, and quantitative reverse transcriptase PCR demonstrated efficient packaging of SIV gRNA by these VLPs. The methodology we employed ensured the availability of >95% pure, biologically active, full-length SIV Pr55^Gag^ which should facilitate future studies to understand protein structure and RNA-protein interactions involved during SIV gRNA packaging.

## 1 Introduction

Retroviruses are present across different species of the animal kingdom and are particularly widespread in mammals, including humans in which they can cause various ailments [1,2]. Some retroviruses cause immunodeficiency syndromes like the human, feline, and simian immunodeficiency viruses (HIV/FIV/SIV), and Mason-Pfizer monkey virus (MPMV), while others are the etiologic agents of a variety of neoplastic diseases, such as avian leukosis and Rous sarcoma viruses (ALV/RSV), human T cell leukemia/lymphoma virus (HTLV), murine, feline, and bovine leukemia viruses (MLV/FLV/BLV), and mouse mammary tumor virus (MMTV) [1,2]. SIV is a member of the lentiviral family of retroviruses and the simian cousin of HIV. SIV is known to cause acquired immunodeficiency syndrome (AIDS) in non-human primates and is believed to have crossed the species barrier into humans, appearing as HIV-1 and 2 [3–5]. The similarities of SIV with HIV makes it an important animal model to study HIV infection and AIDS pathogenesis, especially for the development of HIV vaccines and therapies [3,4].

The retroviral Gag polyprotein serves as the major structural protein that drives virus particle assembly [1,2]. Gag protein has been known to self-assemble into spherical virus-like particles in both *in vitro* systems and in cell culture [[6], [7], [8], [9], [10], [11], [12], [13]]. It has also been widely implicated in the specific encapsidation/packaging of a dimeric retroviral genome in the form of unspliced (full-length) genomic RNA (gRNA) into assembling viral particles, despite the presence of viral spliced RNAs and abundant host RNAs within the cytoplasm of infected cells [[14], [15], [16], [17], [18], [18], [19], [20], [21], [22], [23], [24], [25], [26], [27]]. Specific packaging and/or encapsidation of retroviral gRNA involves the recognition of a particular sequence of the gRNA located at its 5’ end termed “packaging signal” (psi Ψ) which has been shown to assume a higher order RNA structure recognized by the Gag precursor polyprotein [14], [15], [16], [21], [22], [23], [24], [28], [29], [30], [31], [32], [33], [34], [35], [36], [37], [38], [39].

The *gag* gene of SIV encodes its major structural protein, a precursor polypeptide, Pr55^Gag^, which is proteolytically cleaved by the protease enzyme to make mature components of viral particles: NH_2_-p17 (matrix, MA), p27 (capsid, CA), SP1 (spacer peptide 1), p7 (nucleocapsid, NC), SP2 (spacer peptide 2), p6-COOH [40]. Similar to HIV-1, the SIV MA harbors molecular determinants required for Gag to target and associate with the plasma membrane and assist with the incorporation of the envelope (Env) glycoprotein into the budding virions [[41], [42], [43]]. The three-dimensional structure of the SIV MA domain displays remarkable similarity with other retroviruses despite highly divergent primary sequences [44]. The NC protein forms a ribonucleoprotein complex with the dimeric RNA genome in the mature virion and the CA region of Gag forms the protective protein shell around the ribonucleoprotein (NC-RNA) complex [1,2]. The NC region encompasses two zinc finger domains along with several key amino acids that are vital for gRNA packaging and reverse transcription [[45], [46], [47], [48]]. The C-terminal of the SIV Pr55^Gag^ polypeptide contains a p6 domain presenting binding sites for the accessory viral proteins Vpr and Vpx [40], along with components of the endosomal sorting complexes required for transport (ESCRT) associated with viral budding [48,49]. Two short spacer peptides, SP1 and SP2, are also present which separate the CA from the NC and the NC from the p6 domain, respectively. The role of SIV Gag SP1 and SP2 in virus replication has not been investigated thoroughly except that Rauddi and colleagues reported that both the C-terminal domain of SIV capsid and SP1 are crucial for Gag-Gag interactions [13].

Among the domains of the Gag polyprotein, the NC domain is known to be a key determinant of specific recognition of gRNA by the cognate Gag protein, perhaps because of its highly conserved and basic nature as well as the presence of zinc finger motifs that enable RNA-protein interactions [17,21,30,45,[50], [51], [52], [53]]. Furthermore, it has been postulated that NC recognizes retroviral gRNA in a dimeric state given the fact that dimerization is essential for gRNA packaging [54,55]. The NC domain in SIV has been shown to have the same nucleotide binding affinity as HIV-1 and readily displays interactions of NC with the HIV-1 packaging sequences [56,57].

Several recent observations (primarily from HIV-1) have revealed that retroviral RNA packaging is a multidimensional phenomenon in which the full-length Gag polyprotein plays a crucial role during selective recognition and encapsidation of gRNA over host cellular and viral spliced RNAs [19,20,25,27]. For example, previous studies conducted with only the NC domain of Gag suggested that it binds with high affinity to a GGAG tetra loop within stem loop 3 (SL3) of the viral RNA; thus, SL3 was considered the primary packaging domain of HIV-1 [58,59]. In sharp contrast to this, later studies that employed full-length HIV-1 Gag (Pr55^Gag^) revealed that a G//AGG internal loop within SL1 binds to Pr55^Gag^ (instead of the GAGG tetraloop in SL3), enabling its gRNA packaging [19,20,25,27]. Furthermore, it has recently been shown that the p6 domain of HIV-1 Pr55^Gag^ is also crucial for specific binding to gRNA [39]. Therefore, it is imperative to study retroviral gRNA packaging process using full length Gag polyprotein.

Packaging of retroviral gRNA occurs concomitantly with viral assembly, and the viral Gag protein first selectively recognizes gRNA which favors Gag multimerization [1,2]. Viral assembly around a gRNA dimer then requires that Gag selectivity switch from specific to non-specific RNA binding [60,61]. While we and others have identified sequences responsible for SIV gRNA packaging [[62], [63], [64], [65], [66], [67], [68]], how Gag recognizes SIV packaging determinants remains unclear. Furthermore, it also remains unclear whether discrimination between spliced viral RNAs and gRNA is mediated by their initial interactions with Pr55^Gag^, or whether other pathways, such as gRNA nuclear export or its subcellular localization, are also involved, as has been proposed for HIV-1 [37,[69], [70], [71], [72], [73]].

Until recently, limited understanding of retroviral selective encapsidation of the gRNA by assembling retroviral particles has been because of the unavailability of a functionally active full-length Gag which initially interacts with the packaging sequences on the unspliced gRNA. Recent studies have reported purification of full-length Gag of different retroviruses [74–77]. Using these full-length retroviral Gag polyproteins, investigators have identified Gag binding sites on HIV-1, MMTV, and MPMV gRNAs [19,78,79]. This study attempted for the first time to express and purify recombinant full-length SIV Pr55^Gag^ polyprotein using a bacterial protein expression system. Moreover, the recombinant full-length SIV Pr55^Gag^ polyprotein was purified in a native, soluble form without any solubility tags and its functional characteristics were established by both *in vivo* and *in vitro* approaches. The availability of purified SIV Pr55^Gag^ should allow us to explore how full-length Gag is involved in augmenting gRNA packaging by selectively sorting SIV genome over cellular and viral spliced RNAs. Furthermore, such studies are likely to enrich our understanding of the molecular complexities involved during SIV gRNA packaging, especially in defining RNA-protein interactions that take place during SIV replication.

## 2 Results and discussion

### 2.1 SIV Pr55^Gag^-His_6_-tagged protein expression in bacteria

**Fig. 1:**
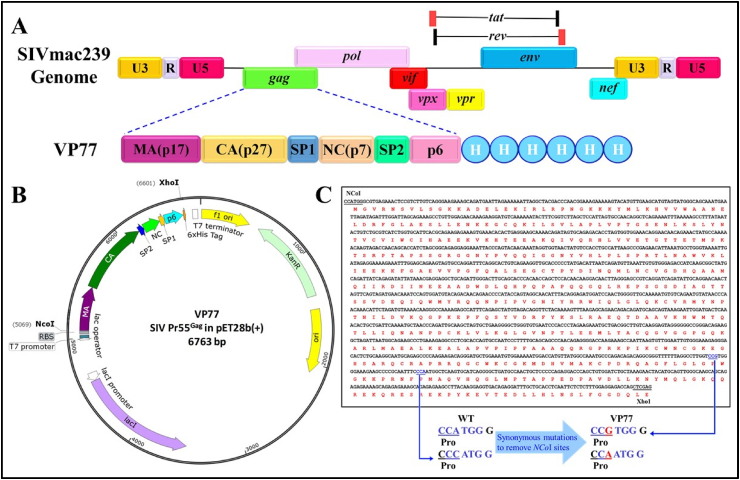
**Construction of VP77 recombinant full-length SIV Pr55**^**Gag**^**bacterial expression vector. (A)** Schematic representation of the SIV genome and SIV full-length Pr55^Gag^ bacterial expression plasmid VP77, with the domain organization of Gag and His_6_-tag. **(B)** Illustration of VP77, the SIV Pr55^Gag^ expression plasmid constructed using pET28b(+) where Pr55^Gag^ is expressed from the bacteriophage T7 promoter. **(C)** The translated sequence of SIV Pr55^Gag^ depicts the synonymous mutations (in red) that were introduced in the sequence to inactivate 2 inherent *Nco*I sites (in blue), for the ease of cloning. (For interpretation of the references to colour in this figure legend, the reader is referred to the Web version of this article.)

**Fig. 2:**
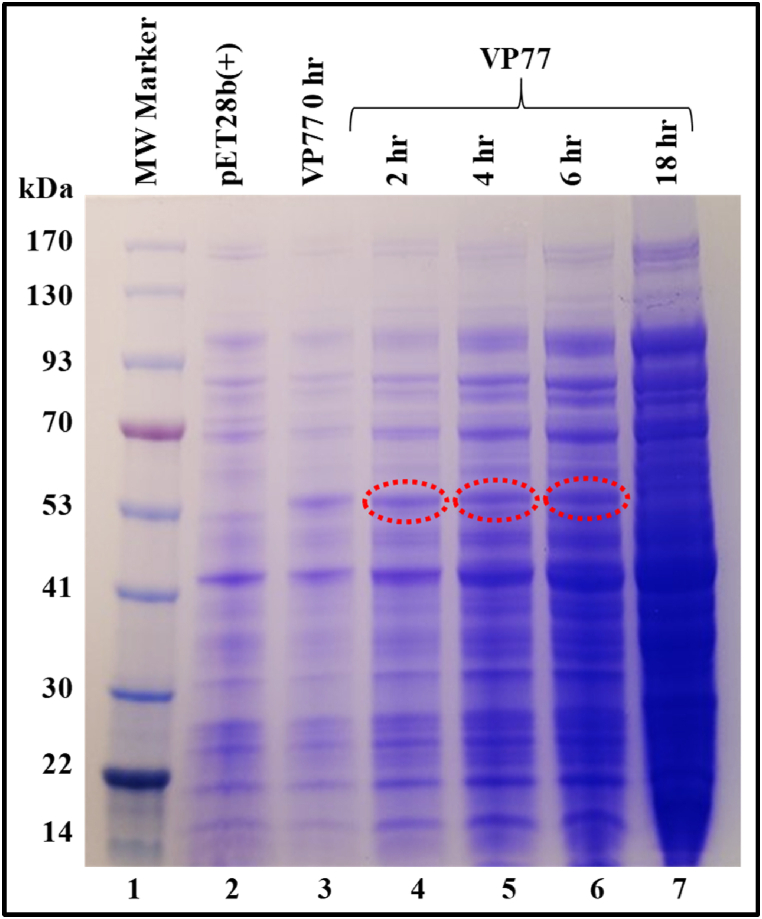
**Expression of the recombinant SIV full-length Pr55**^**Gag**^**-His**_**6**_**-tag fusion protein from E. *coli* total bacterial cell lysates.** Coomassie Brilliant Blue-stained SDS polyacrylamide gel showing expression of recombinant full-length SIV Pr55^Gag^ from total cell lysates at 0, 2, 4, 6 and 18 h after induction with IPTG and cultured at 28 °C. The red dashed circles identify the recombinant protein bands. The uncropped gel image is provided as Supplementary Fig. 1. (For interpretation of the references to colour in this figure legend, the reader is referred to the Web version of this article.)

A pET28b(+) based recombinant bacterial expression plasmid containing the full-length 1.5 kb SIV Pr55 *gag* sequences with a C-terminal hexa-histidine tag was cloned, sequenced and named VP77 (Fig. 1). VP77 was transformed into BL21 bacterial cells for recombinant protein expression. Bacterial cultures were grown suboptimally (at 28 °C) following induction with 0.4 mM IPTG (isopropyl β-d-1-thiogalactopyranoside), required for induction of Gag gene expression. Suboptimal temperature (28 °C) was employed to avoid aberrant assembly of viral proteins in inclusion bodies, as has been reported earlier [8]. Such a strategy has worked very well in our hands for the expression of full-length retroviral Gag proteins from other retroviruses [75–77]. Bacterial cultures were harvested at different time points (0, 2, 4, 6, and 18 h) and protein expression was examined by sodium dodecyl sulfate-polyacrylamide gel electrophoresis (SDS-PAGE) (Fig. 2).

Expression of the recombinant SIV Pr55^Gag^-His_6_-tag was confirmed by Coomassie Brilliant Blue staining as observed by the appearance of a band of ∼55 kDa in size, in a time-dependent manner (lanes 4–6, Fig. 2). BL21 cultures transformed with the empty pET28b(+) expression vector served as the negative control and, the total lysates from pET28b(+) culture showed no protein expression (lane 2, Fig. 2). The low level of protein expression observed at 0 h (prior to induction (lane 3, Fig. 2) can be attributed to the T7 promoter of pET28b(+) vector which has been shown to be leaky in nature [80]. The leaky protein expression did not have any deleterious effect on the bacterial cells as uninterrupted protein expression could be observed in the cells for up to 18 h after induction.

### 2.2 SIV Pr55^Gag^-His_6_-tagged protein expression in the soluble bacterial fraction

**Fig. 3:**
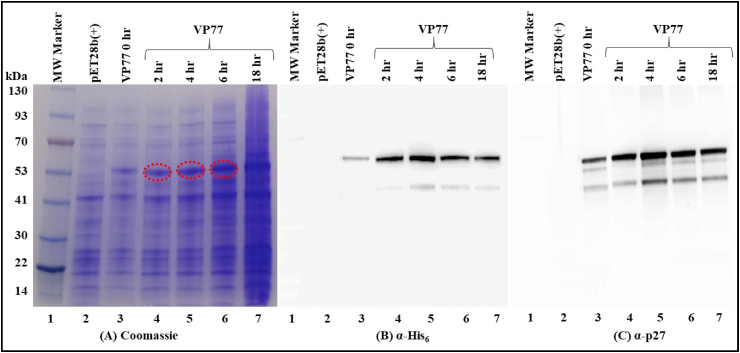
**Recombinant SIV full-length Pr55**^**Gag**^**-His**_**6**_**-tag fusion protein expression in the soluble fraction of E. *coli***. **(A)** SDS-PAGE gel showing recombinant SIV Pr55^Gag^-His_6_-tag fusion protein expression in the soluble fraction at varying time points after suboptimally inducing the bacterial cultures at 28 °C. The red dashed circles identify the recombinant protein bands. Western blot analysis of the soluble fraction confirmed the expression of SIV Pr55^Gag^-His_6_-tag fusion protein at varying time points when analyzed with, **(B)** α-His_6_ and, **(C)** with SIV α-p27 monoclonal antibodies respectively, using equal amounts of protein in each lane. The uncropped gel and western blots are provided in Supplementary Fig. 2. (For interpretation of the references to colour in this figure legend, the reader is referred to the Web version of this article.)

After establishing that the molecular clone VP77 was expressing SIV Pr55^Gag^-His_6_-tagged fusion protein, the next challenge was to determine the solubility of this protein. To establish the solubility of the SIV Pr55^Gag^-His_6_ fusion protein, the SIV Pr55^Gag^-His_6_-tagged protein expression vector (VP77), was transformed into BL21 cells and cultures were harvested at 0, 2, 4, 6 and 18 h following suboptimal induction at 28 °C. CelLytic B buffer supplemented with EDTA-free protease inhibitor, benzonase, and lysozyme, was used to lyse the pelleted bacterial cultures (see Materials and Methods). Following lysis and high-speed centrifugation to pellet down the insoluble material (cell debris and inclusion bodies), the supernatant from these cultures was analyzed by SDS-PAGE. As observed in the total bacterial lysates, the Coomassie Brilliant Blue-stained gel showed a distinct protein band of ∼55 kDa corresponding to SIV Pr55^Gag^-His_6_-tag fusion protein (lanes 4–6, Fig. 3A). Western blotting of the soluble fractions with antibodies against His_6_-tag and SIV α-p27 further confirmed that the protein bands at ∼55 kDa were SIV Pr55^Gag^ with an intact C-terminal His_6_-tag (Fig. 3B and C, respectively). The highest protein expression was observed at 4 h of induction with 0.4 mM IPTG (lane 5, Fig. 3B and C). These results revealed that the SIV Pr55^Gag^-His_6_-tagged protein expressed from VP77 was expressed in soluble bacterial fractions with the highest expression observed 4 h after induction with IPTG.

### 2.3 Identification of an alternative internal initiation codon downstream of a potential Shine-Dalgarno-like ribosome binding site in SIV Gag

**Fig. 4:**
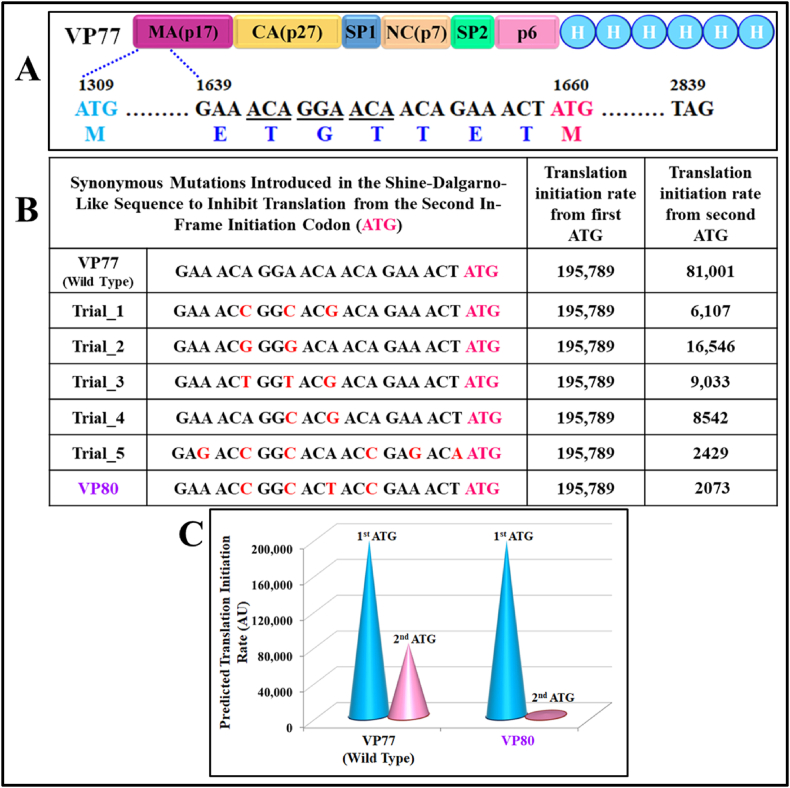
**Synonymous mutations in the Shine-Dalgarno-like sequence to create VP80 recombinant full-length SIV Pr55**^**Gag**^**. (A)** VP77 recombinant full-length SIV Pr55^Gag^ showing the Shine-Dalgarno-like sequence (underlined) which is 9 nucleotides upstream of the second in-frame initiation codon ATG (nucleotide 1660, in pink) and, its corresponding amino acids (shown below the sequence). **(B)** Table listing the predicted translation initiation rates from the actual start codon (in blue) and the second in-frame start codon (in pink) for the wild type VP77 followed by potential **synonymous** mutations (in red) in the region containing the Shine-Dalgarno-like sequence to disrupt it and inhibit translation from the second in-frame start codon. The mutation which yielded the least translation initiation rate from the second start codon was named VP80. **(C)** Graphical comparison of the predicted translation rates from the actual start codon (blue) and, the second in-frame start codon (pink), in the wild type VP77 and mutant VP80 containing the altered Shine-Dalgarno-like sequence in recombinant full-length SIV Pr55^Gag^. (For interpretation of the references to colour in this figure legend, the reader is referred to the Web version of this article.)

Another significant observation made from results shown in Fig. 3 was the presence of a conspicuous protein band with a lower molecular weight of ∼44 kDa (below our target SIV Pr55^Gag^ protein) in all the samples tested (Fig. 3B and C). A similar scenario has been observed previously during the bacterial expression and purification of MMTV Pr77^Gag^-His_6_-tag fusion protein where the lower molecular weight band was found to be due to a second in-frame start codon [75]. Based on these observations, a careful study of the SIV *gag* nucleotide sequence revealed the presence of a second in-frame start codon (ATG) at nucleotide 1660 (351 nucleotides downstream of the native ATG), and another Shine-Dalgarno-like sequence (5’ ACA GGA ACA 3’) 9 nucleotides ahead of this internal ATG, which facilitated the expression of this truncated protein (Fig. 4A).

The UTR Designer, an online software which can predict prokaryotic translation initiation rates (TIR) was employed to further validate our findings [81]. This tool predicted a TIR of 195,789 from the native ATG and a TIR of 81,001 from the second ATG for VP77 (Fig. 4B). A combination of synonymous mutation of the second potential ribosome binding site (Shine-Dalgarno-like sequence) was analyzed using the UTR Designer tool to design VP80, a mutant in which the predicted TIR from the second ATG was reduced to a negligible amount (2073 from 81,001) (Fig. 4B and C).

### 2.4 IMAC (immobilized metal affinity chromatography) purification of bacterially expressed recombinant SIV Pr55^Gag^-His_6_-tagged fusion protein

**Fig. 5:**
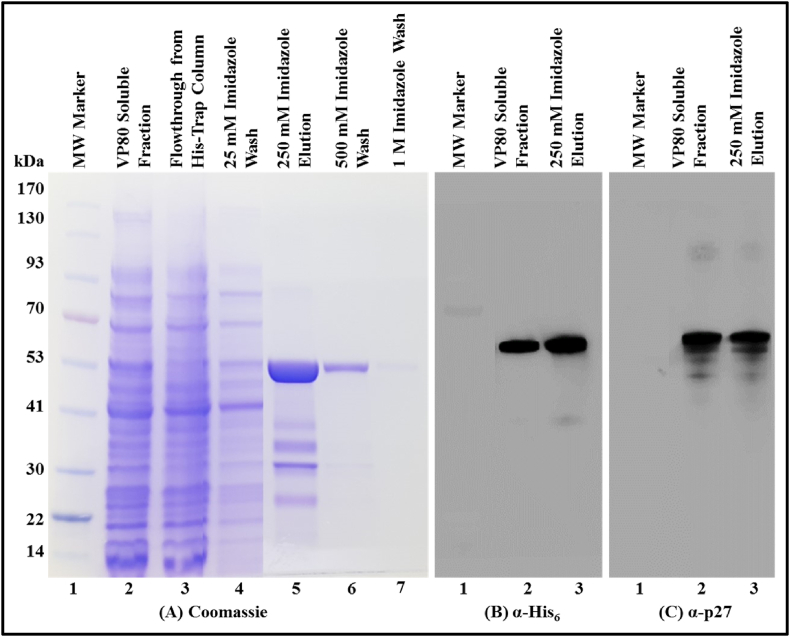
**Immobilized metal affinity chromatography (IMAC) purification of recombinant SIV Pr55**^**Gag**^**-His**_**6**_**-tag fusion protein from the soluble fraction and its Western blot analysis. (A)** Coomassie Brilliant Blue-stained SDS-polyacrylamide gel with fractions obtained during IMAC purification of the VP80 SIV Pr55^Gag^-His_6_-tag protein: lane 2, soluble fraction; lane 3, HisTrap column flow through; lane 4, after wash with 25 mM imidazole buffer; lane 5, after elution using 250 mM imidazole buffer; lane 6, after wash using 500 mM imidazole buffer; lane 7, after wash with 1 M imidazole buffer. Western blot analysis of the soluble fraction and the elute with 250 Mm imidazole buffer using, **(B)** α-His_6_ and, **(C)** SIV α-p27 monoclonal antibodies, respectively, using equal amounts of protein in each lane. The uncropped gel and western blots are provided in Supplementary Fig. 3. (For interpretation of the references to colour in this figure legend, the reader is referred to the Web version of this article.)

Based on the above findings, the synonymously mutated SIV Pr55^Gag^-His_6_-tag fusion protein expression vector (VP80) was constructed and tested for protein expression after transformation into BL21 cells. Protein expression was induced with 0.4 mM IPTG, and transformed cells were grown at 28 °C for 4 h. As expected, immunoblots of the soluble bacterial fraction obtained from this culture upon lysis was incubated with α-His_6_ and SIV α-p27 monoclonal antibodies, individually, to reveal only a distinct band of ∼55 kDa, while the ∼44 kDa band observed earlier was now absent (lane 2, Fig. 5B and C). This confirmed that the synonymous mutations introduced into the Shine-Dalgarno-like sequence in VP80 were effective in inhibiting translation from the second in-frame ATG. Thus, all ensuing experiments were conducted using the molecular clone VP80 containing the synonymously mutated Shine-Dalgarno-like sequence in SIV Pr55 *gag* sequences with a C-terminal hexa-histidine tag in the pET28b(+) protein expression vector.

Next, to purify VP80-expressed SIV Pr55^Gag^-His_6_-tag fusion protein from other proteins present in the bacterial lysate, IMAC was employed (a HisTrap column was connected to the BIO-RAD NGC liquid chromatography system; see Materials and Methods for details). A Coomassie Brilliant Blue-stained gel where the fractions obtained during and after the IMAC purification were run, provides a detailed representation of this process (Fig. 5A). Following bacterial cell lysis, the soluble bacterial fraction was applied to a Ni-immobilized IMAC column under non-denaturing conditions (1 M NaCl). This prevented protein aggregation and precipitation, thereby enhancing protein binding to the column (lane 2, Fig. 5A). The His_6_-tag on the protein bound to the Ni ions on the column; consequently, we observed no protein in the flow through collected from the IMAC column as well as in the column washings using 25 mM imidazole buffer (lanes 3, and 4, Fig. 5A). Following the column wash, the bound protein was eluted using 250 mM imidazole elution buffer (lane 5, Fig. 5A). Subsequent column washes with 500 mM and 1 M imidazole buffers yielded negligible or no protein (lane 6 and 7, Fig. 5A), revealing that almost all of the protein eluted with 250 mM imidazole buffer. Next, the IMAC fraction eluted in 250 mM imidazole buffer was immunoblotted with α-His_6_ and SIV α-p27 monoclonal antibodies, revealing distinct bands corresponding to SIV Pr55^Gag^-His_6_-tag fusion protein (lane 3, Fig. 5B and C). These observations confirmed both the functional inactivation of the Shine-Dalgarno-like sequence and removal of bacterial proteins. Although IMAC is a powerful protein capture step, the protein obtained is often slightly lower in purity; hence, IMAC can be combined with gel filtration chromatography to remove remaining impurities to increase purity of proteins by 95%–99% [82]. We have successfully isolated high purity recombinant MPMV, MMTV and FIV full-length Gag proteins employing this two-step purification methodology [75–77]. Thus, the IMAC-purified protein was subjected to gel filtration chromatography for further purification, as explained in the next section.

### 2.5 Gel filtration chromatography

**Fig. 6:**
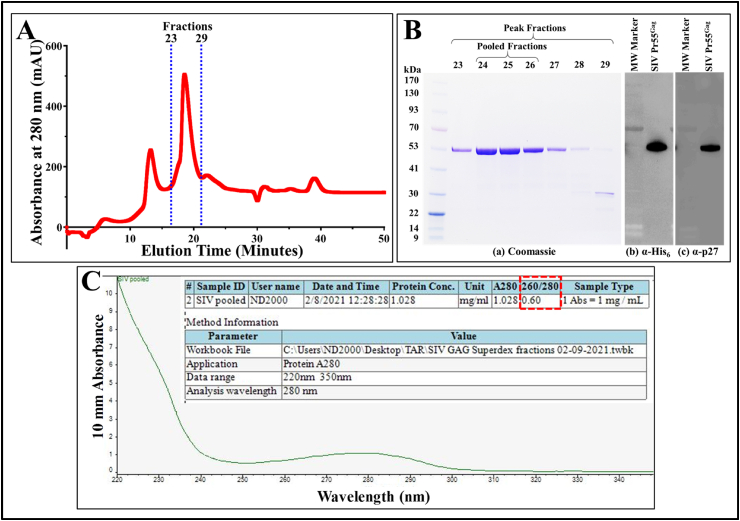
**Analysis of the fractions obtained by size exclusion chromatography of the recombinant SIV Pr55**^**Gag**^**-His**_**6**_**-tag fusion protein. (A)** Chromatogram obtained by plotting the absorbance at 280 nm against the elution time, depicting a distinct peak at between fractions 23 and 29. **(B) (a)** Coomassie Brilliant Blue-stained SDS-PAGE gel of the fractions 23 to 29 showing resolution of the purified recombinant SIV Pr55^Gag^ expressed from VP80. The cleanest fractions (24–26) were pooled and analyzed by western blotting with **(b)** α-His_6_ and, **(c)** SIV α-p27 monoclonal antibodies respectively, using equal amounts of protein. **(C)** Absorbance spectra of the final protein preparation on a 220–350 nm range, depicting a 260/280 ratio of 0.6 highlighted in red box. The uncropped gel and western blots are provided in Supplementary Fig. 4. (For interpretation of the references to colour in this figure legend, the reader is referred to the Web version of this article.)

To proceed with gel filtration, the SIV Pr55^Gag^-His_6_-tagged fusion protein eluted from the IMAC column was further concentrated. Towards this end, Amicon® Ultra 15 centrifugal device (30 kDa molecular weight cut-off) was used to reduce the volume to 1 ml fractions containing ∼2–4 mg/ml protein. Since gel filtration does not depend on binding, it requires small sample volumes (0.5%–4% of the total column volume) at low flow rates to achieve higher resolution [83]. Thus, the filtered and concentrated protein fraction was injected onto a Superdex 200 Increase 10/300 GL column connected to the BIO-RAD NGC liquid chromatography system under non-denaturing conditions. Like the buffers used during IMAC, a high salt concentration (1 M NaCl) was maintained in the buffer during gel filtration to prevent any aggregation or precipitation of the protein on the column. Elution of the protein as 500 μl fractions from the column was monitored by the NGC system based on absorbance at 280 nm. A sharp peak was observed corresponding to fractions 23–29, while an additional smaller peak was observed within the void volume (V_0_) of the column which signifies higher molecular weight aggregates that cannot resolve on the column (Fig. 6A).

Fractions 23–29 were subsequently analyzed by SDS-PAGE to measure their protein purity. The Coomassie Brilliant Blue-stained gel reveals clear protein bands at ∼55 kDa corresponding to the SIV Pr55^Gag^-His_6_-tag fusion protein size (Fig. 6B(a)). The fractions with the highest amount of clean protein (fractions 24–26) were pooled and concentrated using the Amicon® Ultra 15 centrifugal device (30 kDa molecular weight cut-off) to ∼2 mg/ml. Immunoblotting with α-His_6_ and SIV α-p27 monoclonal antibodies demonstrated pure SIV Pr55^Gag^-His_6_-tag fusion protein (Fig. 6B (b) and (c), respectively), thereby, establishing the purity of the pooled fractions. The spectrophotometric absorbance of a protein at 260 nm compared to the value measured at 280 nm is a useful measure to determine the purity of an isolated protein where an ideal 260/280 ratio of 0.6 indicates no nucleic acid contamination in the proteins [84]. Spectrophotometric analysis of the pooled SIV Pr55^Gag^-His_6_-tag fusion protein revealed a A260/A280 ratio of 0.6, indicating purity of greater than 95% (Fig. 6C).

In our hands, a 1.5-L culture of bacteria yielded ∼15 mg of protein after IMAC purification, and only ∼5 mg of the pure SIV Pr55^Gag^-His_6_-tag fusion protein could be recovered, after gel filtration chromatography. These results provide us with an expression and purification system to over-express full-length SIV Pr55^Gag^-His_6_-tag fusion protein of high purity in a soluble form without the use of solubility tags which may confound the interpretations of the downstream applications such as RNA-protein interaction.

### 2.6 *In vitro* assembly of recombinant SIV Pr55^Gag^-His_6_-tagged fusion protein

**Fig. 7:**
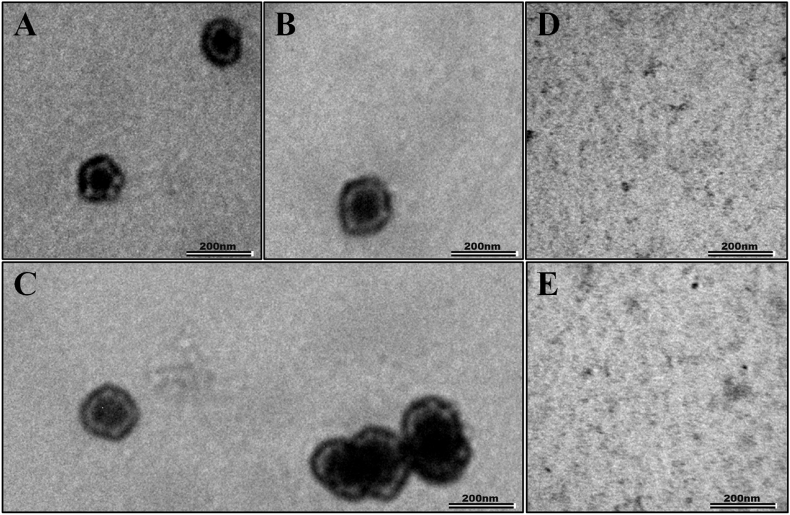
**Transmission electron micrographs showing VLPs assembled *in vitro* by bacterially expressed SIV Pr55**^**Gag**^**-His**_**6**_**-tag fusion protein. (A**–**C)***In vitro* assembled VLPs from grids coated with purified recombinant SIV Pr55^Gag^-His_6_-tag fusion protein in the presence of 4% (*w/w*) yeast tRNA dialyzed overnight at 4 °C at a resolution of 200 nm. **(D**–**E)** Negative control with *in vitro* assembly buffer and purified SIV Pr55^Gag^-His_6_-tag fusion protein dialyzed in the absence of yeast tRNA overnight at 4 °C, at a resolution of 200 nm. The scale bars represent 200 nm.

Subsequent to obtaining purified recombinant SIV Pr55^Gag^-His_6_-tag fusion protein, the next objective was to establish its functional characteristics. Bacterially expressed and purified retroviral Gag proteins have been shown to *in vitro* self-assemble, resulting in virus-like particles (VLPs) in the presence of nucleic acids for most retroviruses, such as HIV-1, FIV, RSV, MPMV and MMTV [10], [12], [74], [75], [76], [77], [85], [86], [87]. Therefore, we tested whether the purified SIV Pr55^Gag^-His_6_-tag fusion protein could self-assemble *in vitro* and form VLPs in the presence of yeast tRNA (4%;*w/w*). Briefly, 2 mg/ml of pure protein in high salt buffer (1 M NaCl) was mixed with 4% (*w/w*) yeast tRNA and dialyzed with a low salt assembly buffer (150 mM NaCl) overnight at 4 °C. The SIV Pr55^Gag^-His_6_-tag fusion protein (2 mg/ml) without yeast tRNA served as the negative control, dialyzed in a similar fashion. Both samples were added on to carbon coated grids, dried, and stained for observation under an electron microscope. The electron micrographs revealed VLPs having a typical spherical appearance with an electron-dense central region ranging approximately 80–100 nm in size (Fig. 7A–C). The size of these immature VLPs corresponded with those that have been reported earlier for HIV-1 and FIV [10,77,86,88,89]. When the *in vitro* assembly was performed without yeast tRNA, the SIV Pr55^Gag^-His_6_-tag fusion protein did not assemble into any VLPs (Fig. 7D–E). These observations confirm that SIV Pr55^Gag^ requires the presence of nucleic acid to self-assemble, as has been established earlier for HIV-1 and FIV [10,12,77,86,88]. This experiment also revealed that SIV Pr55^Gag^-His_6_-tag fusion protein remained biologically functional following a freeze-thaw cycle by retaining its intrinsic property of multimerizing/oligomerizing *in vitro* to assemble into VLPs. Successful *in vitro* assembly of SIV Pr55^Gag^-His_6_-tag fusion protein into immature VLPs suggests that the absence of post translational modifications like myristoylation of the N-terminal glycine residue which have been described for SIV Gag [90] did not adversely affect the assembly properties of bacterially expressed recombinant SIV Pr55^Gag^.

### 2.7 SIV Pr55^Gag^-His_6_-tagged fusion protein expressed in eukaryotic system can form VLPs

**Fig. 8:**
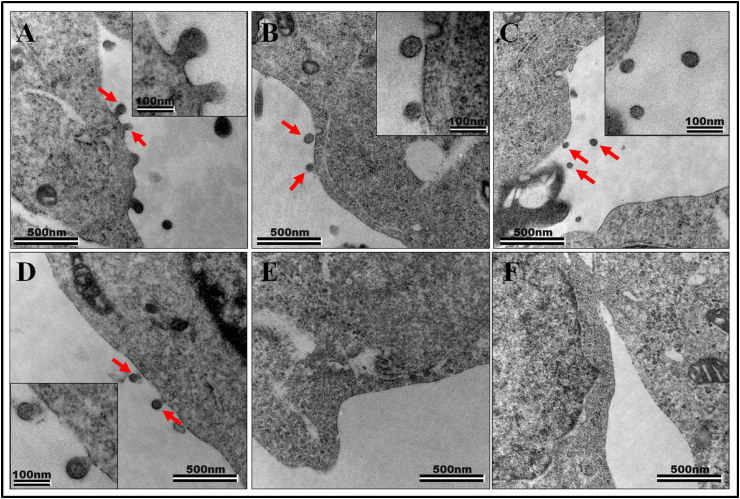
***In vivo* assembly of VLPs in eukaryotic cells by SIV Pr55**^**Gag**^**-His**_**6**_**-tag expression plasmid. (A**–**D)** Electron micrographs showing SIV Pr55^Gag^-His_6_-tag expression plasmid (VP78) transfected into HEK293T cells displaying budding and release of immature VLPs (indicated by red arrows), 72 h post transfection. The electron dense Gag layer is seen accumulating under the plasma membrane and budding out to form VLPs. **(E and F)** The negative control containing only the SIV transfer vector MB41 and no packaging construct showed no budding or release of VLPs. The scale bars used represents 500 nm for the wide-field pictures and 100 nm for the insets. (For interpretation of the references to colour in this figure legend, the reader is referred to the Web version of this article.)

Earlier studies have shown that retroviral Gag or a particular domain of Gag can form VLPs in eukaryotic systems [10,12,[75], [76], [77],^91^]. For example, the MA domain of SIV Gag can form VLPs in eukaryotic cells and such a property of VLP formation could be attributed to the presence of a positively charged domain (residues 26–33) that is conserved in both HIV-1 and SIV. Furthermore, SIV MA when co-expressed with an expression plasmid encoding the Env glycoproteins (gp120 and gp41) could be successfully incorporated on MA VLPs [91]. Therefore, to ascertain whether introduction of the His_6_-tag interferes with VLP production in eukaryotic cells, we created a SIV *gag* eukaryotic expression plasmid, VP78 His(+) containing the native *gag* sequence with a His_6_-tag at its C-terminus and without synonymously mutating the putative Shine-Dalgarno-like sequence in front of the second in frame Gag start site. The constitutive transport element (CTE) from MPMV was added to this eukaryotic expression plasmid downstream of the SIV *gag* sequence to enable proper export and translation of the respective mRNA from the nucleus to the cytoplasm [92]. HEK293T cells were transfected with VP78, while cells transfected with only the SIV transfer vector MB41 and no packaging construct served as the negative control (mock). Seventy-two hours post transfection, the transfected cells were trypsinized and processed for visualization of VLP production by TEM. As shown in Fig. 8A–D, electron dense regions could be observed at the plasma membrane from where VLPs were budding out. Such budding VLPs were absent in the negative control (Fig. 8E–F). Most of the particles observed were within the 80–100 nm size which is consistent with earlier reports for immature SIV Gag VLPs without the envelope [93,94]. These findings demonstrate that SIV Pr55^Gag^ having a C-terminal His_6_-tag did not interfere with Gag expression or its ability to form VLPs in eukaryotic cells.

### 2.8 VLPs produced by SIV Pr55^Gag^-His_6_-tagged fusion protein in eukaryotic cells can package SIV unspliced, sub-genomic RNA efficiently

**Fig. 9:**
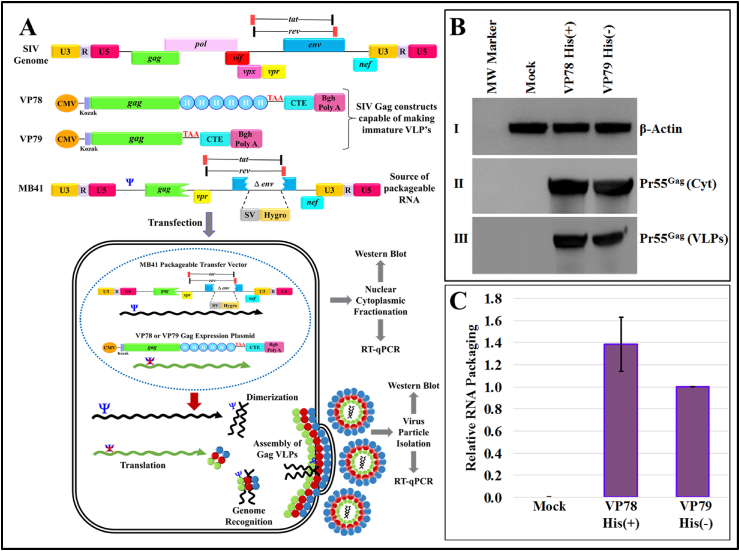
**Two-plasmid genetic complementation assay depicting formation of VLPs in eukaryotic cells by SIV Pr55**^**Gag**^**with and without His**_**6**_**-tag. (A)** Schematic representation of the SIV genome, SIV full-length eukaryotic expression constructs VP78 and VP79, expressing Pr55^Gag^ with and without His_6_-tag and, the SIV transfer vector MB41 which serves as a source for the packageable RNA. Illustration of the *in vivo* two-plasmid genetic complementation assay where the HEK293T producer cells are co-transfected with the SIV sub-genomic transfer vector (MB41) and, the recombinant SIV Pr55^Gag^ expression plasmids (VP78 or VP79). These cells then produce VLPs containing MB41-specific RNA due to the presence of the packaging signal (Ψ) on the transfer vector. The cytoplasmic fractions and pelleted VLPs were analyzed for Gag expression by western blotting and transfer vector RNA expression by RT-qPCR. **(B)** Panels I and II are immunoblots of the cytoplasmic fraction from HEK293T producer cells probed with α-β actin monoclonal antibody at 1:25000 dilution and, SIV α-p27 monoclonal antibody used at 1:100 dilution, respectively. Panel III is the immunoblot of the ultracentrifuged VLPs probed with SIV α-p27 monoclonal antibody at 1:100 dilution. **(C)** Relative RNA packaging efficiency between the VP78 His(+) and VP79 His(−) SIV Pr55^Gag^ RNAs respectively, obtained after RT-qPCR. The relative RNA packaging efficiency was determined by dividing viral RNA packaging values to the cytoplasmic expression normalized to secreted alkaline phosphatase (SEAP) expression for the respective clones. The difference in packaging efficiency between the two clones was statistically not significant. Mock contains only the transfer vector and no packaging construct; hence, it cannot be packaged into viral particles and cannot be picked upon western blots. The uncropped western blots are provided in Supplementary Fig. 5.

Next, we tested the potential of the recombinant SIV Pr55^Gag^-His_6_-tag fusion protein to package SIV RNA. To explore this, the full-length SIV Gag eukaryotic expression plasmids, VP78 containing His_6_-tag (His+) and VP79 without His_6_-tag (His-), were created (Fig. 9A). This was necessary to ensure that the presence of His_6_-tag did not interfere with the nucleic acid binding ability of SIV Pr55^Gag^ during gRNA packaging *in vivo*, as has been suggested earlier in the case of HIV while performing *in vitro* biochemical assays [95]. A two-plasmid genetic complementation assay was developed using these plasmids to express SIV Pr55^Gag^ and make VLPs with and without His_6_-tag, and the SIV transfer vector MB41 [65] which serves as the source of packageable RNA, as shown in Fig. 9A.

Briefly, HEK293T cells were co-transfected individually with either VP78 His(+) or VP79 His(−) constructs along with MB41, while cells transfected with only the SIV transfer vector MB41 and no Gag expression plasmids served as the negative control (mock). In order to ensure viral particles were produced by both His(+) and His(−) transfer vectors, immunoblotting was performed using SIV α-p27 monoclonal antibody on the cell lysates prepared from both the transfected cultures and the viral particles pelleted from the transfected supernatants. The immunoblots revealed similar levels of expression of SIV Pr55^Gag^ in the cytoplasm (Fig. 9B; Panel II) and successful formation of VLPs by both the SIV Pr55^Gag^ His(+) and His(−) constructs (Fig. 9B; Panel III).

Next, RNAs were extracted from both the transfected cultures (cytoplasmic fractions) and VLPs harvested from transfected cultures. DNase-treatment of the RNA preparations was performed followed by PCR with MB41 vector-specific primers (OTR1650 and OTR1651) to demonstrate the absence of any contaminating plasmid DNA from the transfected cultures (data not shown). The DNase-treated RNAs were used to make cDNAs and tested for their relative packaging efficiency (RPE) into the VLPs produced by the SIV Pr55^Gag^ His(+) and His(−) expression constructs using our validated SYBR-Green RT-qPCR assay [68]. As can be seen from Fig. 9C, both the His(+) and His(−) SIV Gag expression constructs (VP78 and VP79, respectively), could successfully package the SIV transfer vector RNA from MB41 with VP78 His(+) showing a slightly better RNA packaging efficiency than VP79 His(−) which was not statistically significant (*p* value = 0.1121; Fig. 9C). We attribute this statistically insignificant increase in efficiency to the addition of the positively charged His_6_-tag at the C-terminal of SIV Pr55^Gag^ which may have increased the basic nature of the polyprotein and stabilized its interaction with the RNA, as has been suggested earlier for MPMV Pr78^Gag^ and MMTV Pr77^Gag^ [75,76]. These data confirmed that when full-length recombinant SIV Pr55^Gag^ containing C-terminal His_6_-tag was expressed, it did not affect its biological activity and thus, could efficiently form VLPs with the ability to encapsidate SIV transfer vector RNA.

## 3 Conclusions

We report successful cloning, expression, and purification of recombinant full-length SIV Pr55^Gag^ with a C-terminal His_6_-tag using a prokaryotic expression system. The protein expressed was present in the soluble fraction without employing any solubility tags which are generally bulky proteins or protein domains that may interfere with downstream applications such as RNA-interaction or conformational studies. The identity of the purified protein was established using specific SIV α-p27 as well as α-His_6_ monoclonal antibodies. The SIV Pr55^Gag^-His_6_-tag fusion protein was able to form VLPs *in vitro*, while the VLPs formed in the eukaryotic cells were functionally able to encapsidate SIV transfer vector RNA despite the presence of the His_6_-tag. The ability to purify and express large quantities of recombinant SIV Pr55^Gag^ should facilitate its structural and functional studies, especially those related to understanding the intricacies involved in RNA-protein interactions during SIV gRNA packaging. The availability of recombinant SIV Pr55^Gag^ should pave the way to perform biochemical studies, such as band-shift and footprinting assays, to learn where on SIV gRNA Gag binds during gRNA packaging as we and others have recently shown for other retroviruses [19,20,25,27,78,79]. Studies using full-length Gag should prevent ambiguities in identifying Gag binding sites on the gRNA that could be missed while using proteins expressed from truncated or partial Gag, such as the NC domain only. Furthermore, many functional aspects of the Gag proteolytic processing and conformational changes in Gag that occur during virus maturation and its oligomerization can now be investigated in depth with the availability of this biologically active and functional SIV Pr55^Gag^. Since the protein obtained is >95% pure, this makes it ideal for applications such as drug interaction studies using peptides as pharmaceuticals, structure-function relationship studies, and for the study of protein structure by cryo-electron microscopy. One of the limitations of our study could be the absence of post translational modifications in the bacterially expressed recombinant SIV Pr55^Gag^. Native SIV Pr55^Gag^ undergoes two major post translational modifications, namely proteolytic cleavage of precursor Gag into its subunits after budding [96], and N-terminal myristoylation of the glycine residue which targets Gag towards the plasma membrane for proper assembly [90]. Another possible limitation of the study could be that we did not add zinc during SIV Pr55^Gag^ purification and/or during *in vitro* assembly of VLPs which may be important for proper folding of the NC domain of Gag since it has two zinc finger binding domains. However, successful *in vitro* assembly of VLPs demonstrates that bacterially expressed recombinant SIV Pr55^Gag^ can assemble properly and thus, is optimal for the above-mentioned studies without these potential caveats.

## 4 Materials and methods

### 4.1 Nucleotide numbers

Designation of nucleotides are based on the GenBank accession number M33262 for SIVmac239 [3].

### 4.2 Construction of full-length SIV Pr55^Gag^ prokaryotic expression plasmids

The complete open reading frame (ORF) of SIV full-length Gag (Pr55^Gag^; nucleotides 1309–2839) was chemically synthesized (Macrogen, South Korea) containing restriction enzyme sites namely *Nco*I at the 5’and *Xho*I at the 3’ end. For the ease of cloning, two inherent *Nco*I sites (CCATGG) in the Gag ORF at nucleotide positions 2623 and 2651 were modified to CCGTGG and CAATGG, respectively, to inactivate the *Nco*I sites while maintaining the native amino acid proline at both locations (Fig. 1C). The chemically synthesized full-length Gag ORF and the bacterial expression vector pET28b(+) were cleaved with *Nco*I and *Xho*I restriction enzymes and ligated together to generate clone VP77. Thus, VP77 expressed an in-frame recombinant fusion protein comprising of full-length SIV Pr55^Gag^ and a hexa-histidine tag at its C-terminus (Pr55^Gag^-His_6_-tag) as shown in Fig. 1, with a predicted molecular weight of 58.1 kDa calculated by ExPASy-pI/Mw tool [97]. Preliminary expression of the SIV Pr55^Gag^ revealed the presence of a Shine-Dalgarno-like sequence (5’ ACAGGAACA 3’) 9 nucleotides ahead of a second in-frame start codon (ATG) located at nucleotide 1660, resulting in the expression of a shortened N-terminal protein ∼44 kDa in size. To inhibit the expression of this truncated N-terminal protein, VP77 was further modified by introducing synonymous mutations in the region (5’ GAA ACA GGA ACA ACA GAA ACT *ATG* 3’) spanning the Shine-Dalgarno-like sequence (underlined) till the second in-frame ATG (italics). Different combinations of mutations were introduced and using the UTR Designer tool [81], the translation initiation rates from the second start codon were predicted. The minimal synonymous mutations that gave the least translation initiation rate from the second in-frame ATG was selected for chemical synthesis. The bacterial expression plasmid pET28b(+) was used to clone this chemically-synthesized DNA to create VP80 containing the modified Shine-Dalgarno-like sequence (mutated nucleotides shown in bold; 5’ GAA AC**C** GG**C** AC**T** AC**C** GAA ACT *ATG* 3’). To ensure that SIV Pr55^Gag^ was in-frame with an intact His_6_-tag, VP77 and VP80 clones were confirmed by sequencing.

### 4.3 Construction of full-length SIV Pr55^Gag^ eukaryotic expression plasmids

Eukaryotic expression plasmids expressing full-length SIV Pr55^Gag^ with and without His_6_-tag were also created. Using the prokaryotic expression plasmid VP77 as the template, PCR amplification was performed using oligo set OTR1549 and OTR1550 to create SIV Pr55^Gag^ with His_6_-tag, and oligo set OTR1549 and OTR1551 to create SIV Pr55^Gag^ without His_6_-tag. OTR1549 (5’ ccg *CTC GAG* GCC GCC ACC **ATG** GGC GTG AGA AAC TCC GTC 3’) was the sense oligo containing 3 dummy nucleotides (lowercase) followed by an *Xho*I site (italicized) and a Kozak sequence (underlined) just upstream of Gag initiation codon (bold). OTR1550 (5’ T TCT CTC TTT GGA GGA GAC CAG CAC CAC CAC CAC CAC CAC **TAG** *CTC GAG* cgg 3’) was the anti-sense oligo containing the SIV *gag* sequences followed by His_6_-tag sequence (underlined), termination codon (bold), an *Xho*I site (italicized) and 3 dummy nucleotides (lowercase). OTR1551 was another anti-sense oligo (5’ T CTC TTT GGA GGA GAC CAG **TAG** *CTC GAG* cgg 3’) containing SIV *gag* sequences followed by the termination codon (bold), an *Xho*I site (italicized) and 3 dummy nucleotides (lowercase). The Phusion High-Fidelity PCR Kit (New England Biolabs) was used for PCR amplification with its standard PCR conditions and primer annealing at 61 °C for 30 s. The PCR-amplified products with and without His_6_-tag were cleaved with *Xho*I restriction endonuclease and cloned into a eukaryotic expression plasmid (pcDNA3) that had already been cleaved with *Xho*I to generate VP78 and VP79, respectively. In order to facilitate efficient SIV Gag mRNA nuclear export and translation, the constitutive transport element (CTE) from MPMV [92,[98], [99], [100], [101]] was cloned immediately after the *gag* stop codon, as has been reported earlier [75–77]. The resulting clones, VP78 and VP79, were confirmed by sequencing. Details of the primer pairs used for introducing specific mutations and/or for cloning are listed in Supplementary Table 1. The specific conditions employed during PCR cycles as well as complete details of cloning are available from the authors upon request.

### 4.4 Growth media and bacterial strains used for cloning and protein expression

During the course of cloning, the ligated DNAs were introduced into the bacteria by transforming DH5α strain of E. *coli* using the standard heat shock protocol in the presence of required antibiotics (kanamycin; 50 μg/ml and/or ampicillin; 100 μg/ml) in Luria-Bertani (LB) media, as described earlier [75–77]. For bacterial protein expression, the prokaryotic recombinant protein clones VP77 and VP80 were transformed into T7 Express (New England Bio Labs), a BL21 derivative of E. *coli*. LB medium containing 50 μg/ml of kanamycin was used to culture a single colony following transformation, as reported earlier [75–77].

### 4.5 Large scale expression of recombinant SIV Pr55^Gag^-His_6_-Tagged fusion protein in bacteria

An isolated colony from transformed BL21 cells was used to inoculate 50 ml of LB media containing appropriate antibiotic (kanamycin 50 μg/ml) and grown overnight at 37 °C while shaking at 200 revolutions per minute (rpm). Next morning, 2-L baffled Erlenmeyer flasks containing 500 ml LB with 50 μg/ml kanamycin were inoculated with this overnight culture and grown at 28 °C until the optical density at 600 nm (OD600) reached approximately 0.6. This was followed by 0.4 mM IPTG induction and the culture was allowed to grow for an additional 4 h at 28 °C, as described previously [75–77]. At 4 h, the bacterial culture was centrifuged at 6300×*g* for 15 min at 4 °C and the pellets were frozen at −80 °C for monitoring protein expression and further purification.

### 4.6 Recombinant SIV Pr55^Gag^-His_6_-tagged fusion protein purification by IMAC and size exclusion chromatography

The recombinant SIV Pr55Gag-His_6_-tagged fusion protein was purified as has been reported earlier for MPMV, MMTV, and FIV [75–77]. Briefly, to lyse the bacterial pellets, ice cold CelLytic B buffer (Sigma-Aldrich, USA) was used after adding EDTA-free protease inhibitor, lysozyme, and benzonase. The lysed cells were centrifuged at 48,000×*g* for 1 h at 4 °C to collect the soluble fraction of the protein which was mixed with a 4X binding buffer (4.0 M NaCl, 0.2 M Tris-HCl of pH 8.0, 100 mM imidazole, 40 mM β-mercaptoethanol, 10 mM dithiothreitol (DTT), 0.4% (*w/v*) Tween-20) to get a final concentration of 1X. The diluted lysate was filtered and applied to a HisTRAPTM FF 5 ml column attached to the BIO-RAD NGC liquid chromatography system and equilibrated with 1X equilibration buffer (1 M NaCl, 50 mM Tris-HCl (pH 8.0), 25 mM imidazole, 10 mM β-mercaptoethanol, 2.5 mM DTT, 0.1% (*w/v*) Tween-20 and 10% (*v/v*) glycerol). After applying the lysate onto the column, it was washed with 5 column volumes of the same equilibration buffer containing 25 mM imidazole. The bound His_6_-tagged protein was then eluted using 5 column volumes of elution buffer which is the same equilibration buffer containing 250 mM imidazole. The column was further washed with 500 mM and 1 M imidazole containing buffers to ensure complete removal of bound protein. Amicon® Ultra 15 (30,000 molecular weight cut-off membrane) column was used to concentrate the eluted protein and the quality of the protein was analyzed by SDS-PAGE followed by staining with Coomassie Brilliant Blue and immunoblotting. To further purify the protein to homogeneity, size exclusion chromatography was employed. The concentrated protein was injected onto the Superdex 200 Increase 10/300 GL column attached to the BIO-RAD NGC liquid chromatography system as 1 ml fractions at a concentration of 2 mg/ml. The column was equilibrated with 1X gel filtration buffer (50 mM Tris-HCl (pH 8.0), 1 M NaCl, 1 mM DTT) and the protein was eluted in the same buffer. The resolved protein fractions that gave a peak were further evaluated by SDS-PAGE and the cleanest fractions were pooled and concentrated into 2 mg/ml aliquots which were flash frozen and stored at −80 °C. Purity of the pooled fractions of SIV Pr55^Gag^-His_6_-tagged fusion protein was determined by measuring the 260/280 nm absorbance ratio.

### 4.7 SDS-PAGE and western blotting

The expression and purity of bacterially expressed SIV Pr55^Gag^-His_6_-tagged fusion protein was monitored by SDS-PAGE and western blotting, as described previously using α-SIV p27 CA monoclonal antibody (ABL, USA; 1:1000 dilution) and α-His_6_-horseradish peroxidase (HRP)-conjugated monoclonal antibody (Sigma-Aldrich; 1:4000 dilution).

### 4.8 *In vitro* assembly of SIV Pr55^Gag^-His_6_-Tagged fusion protein to form VLPs

To study the ability of SIV Pr55^Gag^-His_6_-tagged recombinant fusion protein to form VLPs, 200 μl of 2 mg/ml of the protein (in 1 M NaCl, 50 mM Tris-HCl (pH 8.0), 1 mM DTT) was added to yeast tRNA at a protein to RNA ratio of 4% (*w/w*). This protein-tRNA mixture was allowed to dialyze into a buffer containing 150 mM NaCl, 50 mM Tris (pH 8.0), and 10 mM DTT overnight at 4 °C. This was followed by spotting 8 μl of the sample on to a carbon-coated formvar grid which was stained by uranyl acetate and used for TEM, as described previously [75–77].

### 4.9 Visualization of SIV VLPs produced in eukaryotic cells by SIV Pr55^Gag^-His_6_-Tagged fusion protein

To visualize VLPs produced by the SIV Pr55^Gag^-His_6_-tagged fusion protein using TEM, an expression plasmid expressing this protein was transfected into human embryonic kidney HEK 293T cells which were processed as described previously [77]. Briefly, approximately 72 h post transfection, cells were harvested via trypsinization, pelleted, and fixed in Karnovsky’s fixative. Sections (ultrathin; 95 nm) of the resin embedded samples were stained for TEM using 1% osmium tetroxide, as described previously and visualized using TEM (FEI Tecnai Biotwin Spirit G2) [77].

### 4.10 Eukaryotic expression of SIV Pr55^Gag^-His_6_-Tagged fusion protein

Eukaryotic expression was monitored by transfecting HEK293T cells with the pcDNA3-based SIV Pr55^Gag^ eukaryotic expression plasmids VP78 (His+) and VP79 (His-), as described in our recent studies [75–77]. To isolate VLPs, ∼72 h after transfection, media from the transfected cells was harvested and subjected to ultracentrifugation. Isolated VLPs were then used for RNA extraction and western blotting.

### 4.11 Reverse transcriptase PCR (RT-PCR) and RT-qPCR to estimate relative packaging efficiency (RPE) of SIV gRNA

The efficiency of SIV RNA being packaged into the recombinant Gag VLPs was estimated using RT-qPCR. For this purpose, a customized RT-qPCR was developed that was based on SYBR Green chemistry that measured the amount of SIV subgenomic RNA expressed from the SIV transfer vector (MB41, [65]. This employed two newly designed oligos (OTR1650/OTR1651; Supplementary Table 1) at 300 nM. PCR was conducted using the SYBR Green qPCR master mix from Solis BioDyne (5x Hot FirePol EvaGreen qPCR Supermix). Expression of the SIV-specific signal was quantified using the 2^−ΔΔCT^ method, as described previously [68], using β-actin as the endogenous control (OFM456/OTR1199; Supplementary Table 1) at a concentration of 500 nM. Briefly, the most suitable primer concentration was established by testing various primer concentrations that provided maximal amplification with a single, sharp peak following melt curve analysis. The samples were tested in triplicates using the Applied Biosystems 7500 ABI QuantStudioTM7 Flex System (Applied Biosystems, USA) for 40 cycles at an annealing temperature of 60 °C. The relative packaging efficiency (RPE) of SIV gRNA by each of the SIV Pr55^Gag^ constructs was quantified by estimating the ratio of RNA packaged into the virus particles relative to its cytoplasmic expression normalized to the secreted alkaline phosphatase (SEAP) expression levels for each sample (RPE = Virion RNA content/SEAP-normalized Cyt RNA expression). The significance of the results was expressed in *p* values using the student’s t-test.

## Production notes

### Author contribution statement

Vineeta N. Pillai, Lizna Mohamed Ali: Performed the experiments; Analyzed and interpreted the data; Wrote the paper.

Suresha G. Prabhu, Anjana Krishnan: Performed the experiments.

Saeed Tariq: Performed the experiments; Contributed reagents, materials, analysis tools or data.

Farah Mustafa, Tahir A. Rizvi: Conceived and designed the experiments; Analyzed and interpreted the data; Contributed reagents, materials, analysis tools or data; Wrote the paper.

### Funding statement

Prof Tahir A. Rizvi was supported by College of Medicine and Health Sciences, United Arab Emirates University [NP-18-30/31M388; NP-20-29/31M476/12M040], ASPIRE [21M150], Zayed Bin Sultan Center for Health Sciences, United Arab Emirates University [UCBR-31R143].

### Data availability statement

Data included in article/supp. material/referenced in article.

### Declaration of interest’s statement

The authors declare no competing interests.
