# Supplementary material for: Expression, purification, and functional characterization of soluble recombinant full-length simian immunodeficiency virus (SIV) Pr55Gag
Source: Heliyon. 2023 Jan 10;9(1):e12892. doi: 10.1016/j.heliyon.2023.e12892 (PMC9853374; doi:10.1016/j.heliyon.2023.e12892)
Supplement: Multimedia component 1 [file mmc1.pptx]

## Slide 1
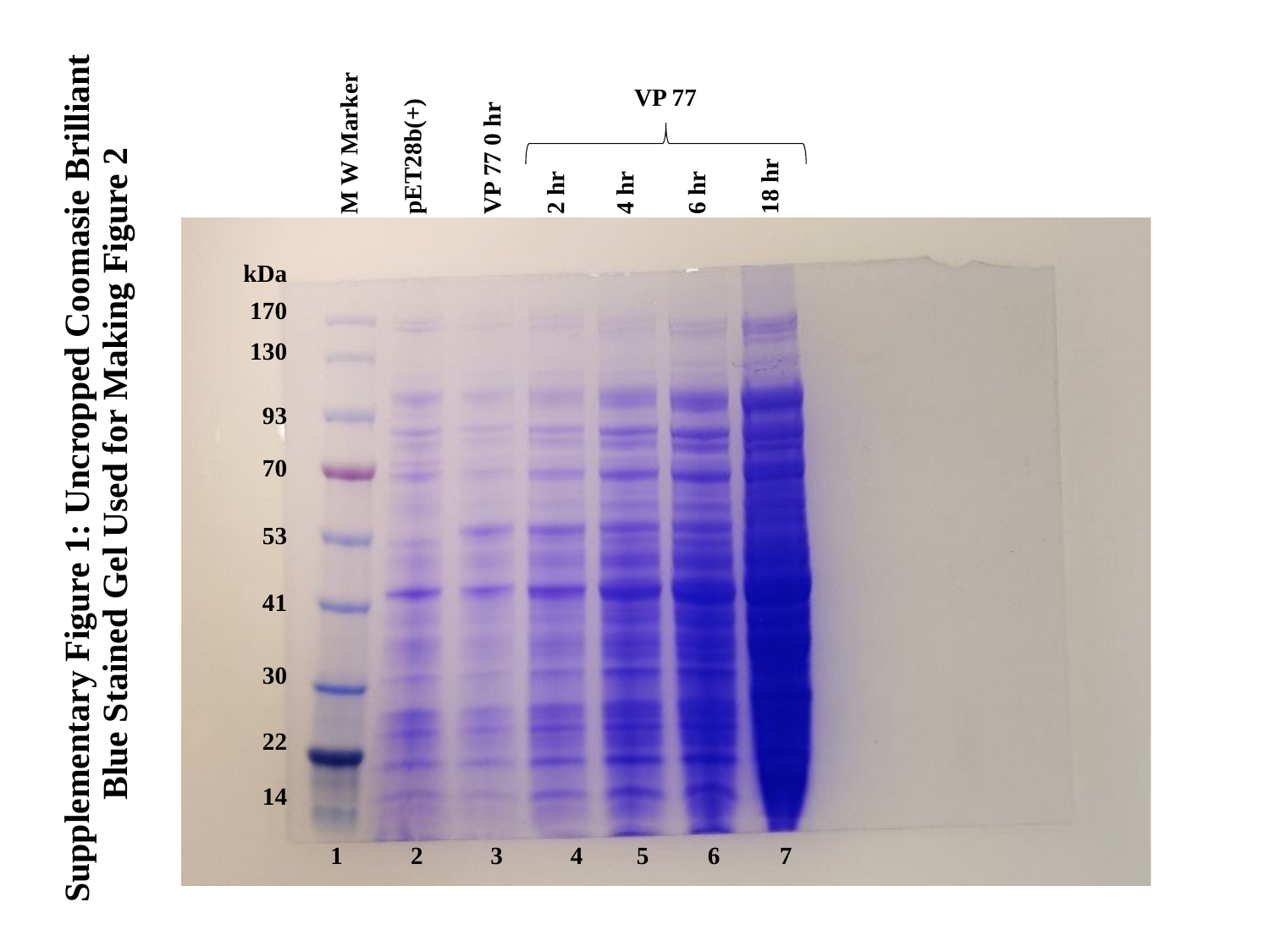

VP 77
M W Marker
VP 77 0 hr
pET28b(+)
2 hr
4 hr
6 hr
18 hr
 kDa
170
130
93
70
53
41
30
22
14
# Supplementary Figure 1: Uncropped Coomasie Brilliant Blue Stained Gel Used for Making Figure 2
1
2
3
4
5
6
7
