# Supplementary material for: Expression, purification, and functional characterization of soluble recombinant full-length simian immunodeficiency virus (SIV) Pr55Gag
Source: Heliyon. 2023 Jan 10;9(1):e12892. doi: 10.1016/j.heliyon.2023.e12892 (PMC9853374; doi:10.1016/j.heliyon.2023.e12892)
Supplement: Multimedia component 2 [file mmc2.pptx]

## Slide 1
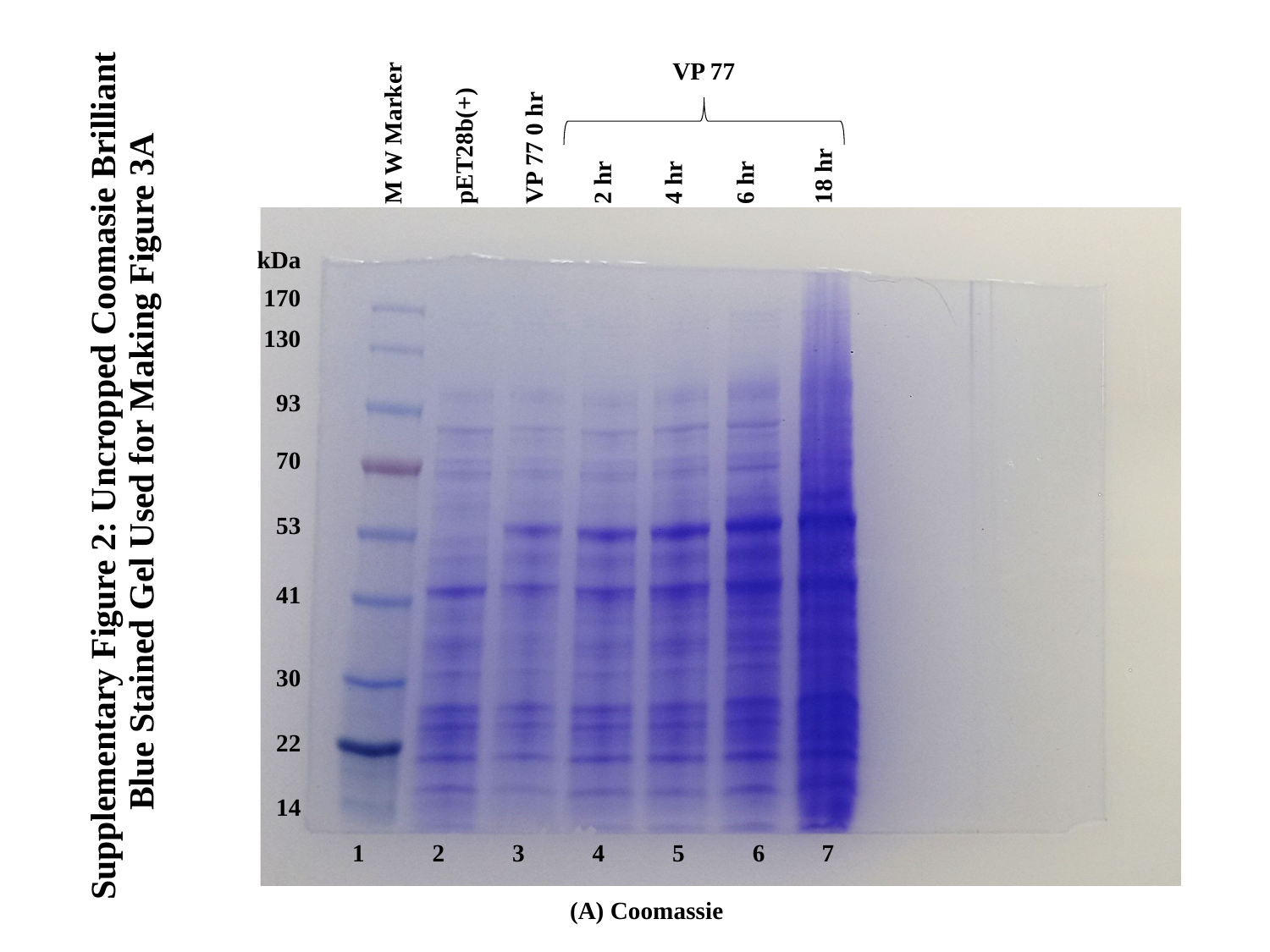

VP 77
M W Marker
VP 77 0 hr
pET28b(+)
2 hr
4 hr
6 hr
18 hr
 kDa
170
130
93
70
53
41
30
22
14
# Supplementary Figure 2: Uncropped Coomasie Brilliant Blue Stained Gel Used for Making Figure 3A
1
2
3
4
5
6
7
(A) Coomassie

## Slide 2
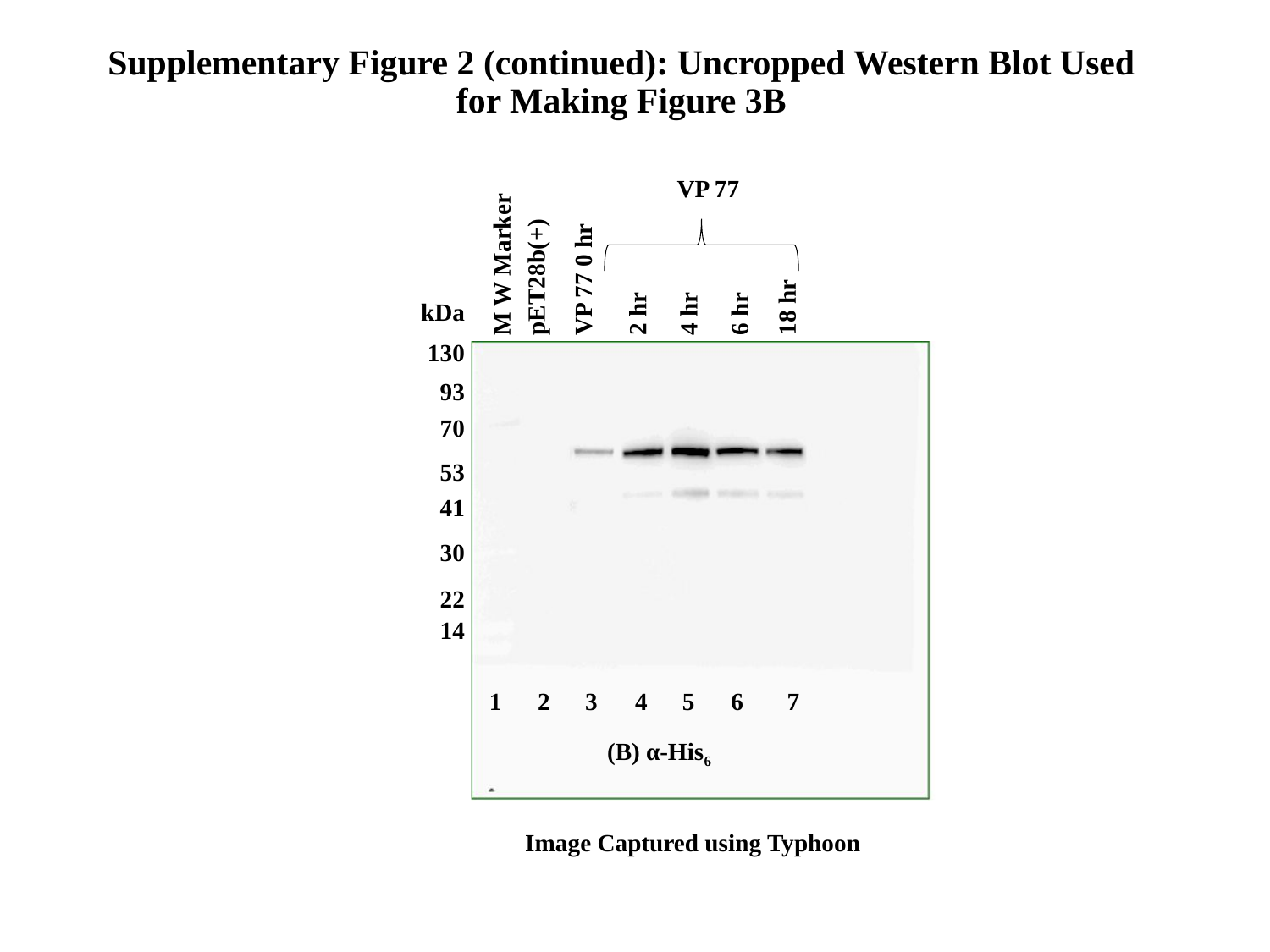

Supplementary Figure 2 (continued): Uncropped Western Blot Used for Making Figure 3B
VP 77
M W Marker
VP 77 0 hr
pET28b(+)
2 hr
4 hr
6 hr
18 hr
 kDa
130
93
70
53
41
30
22
14
1
2
3
4
5
6
7
(B) α-His6
Image Captured using Typhoon

## Slide 3
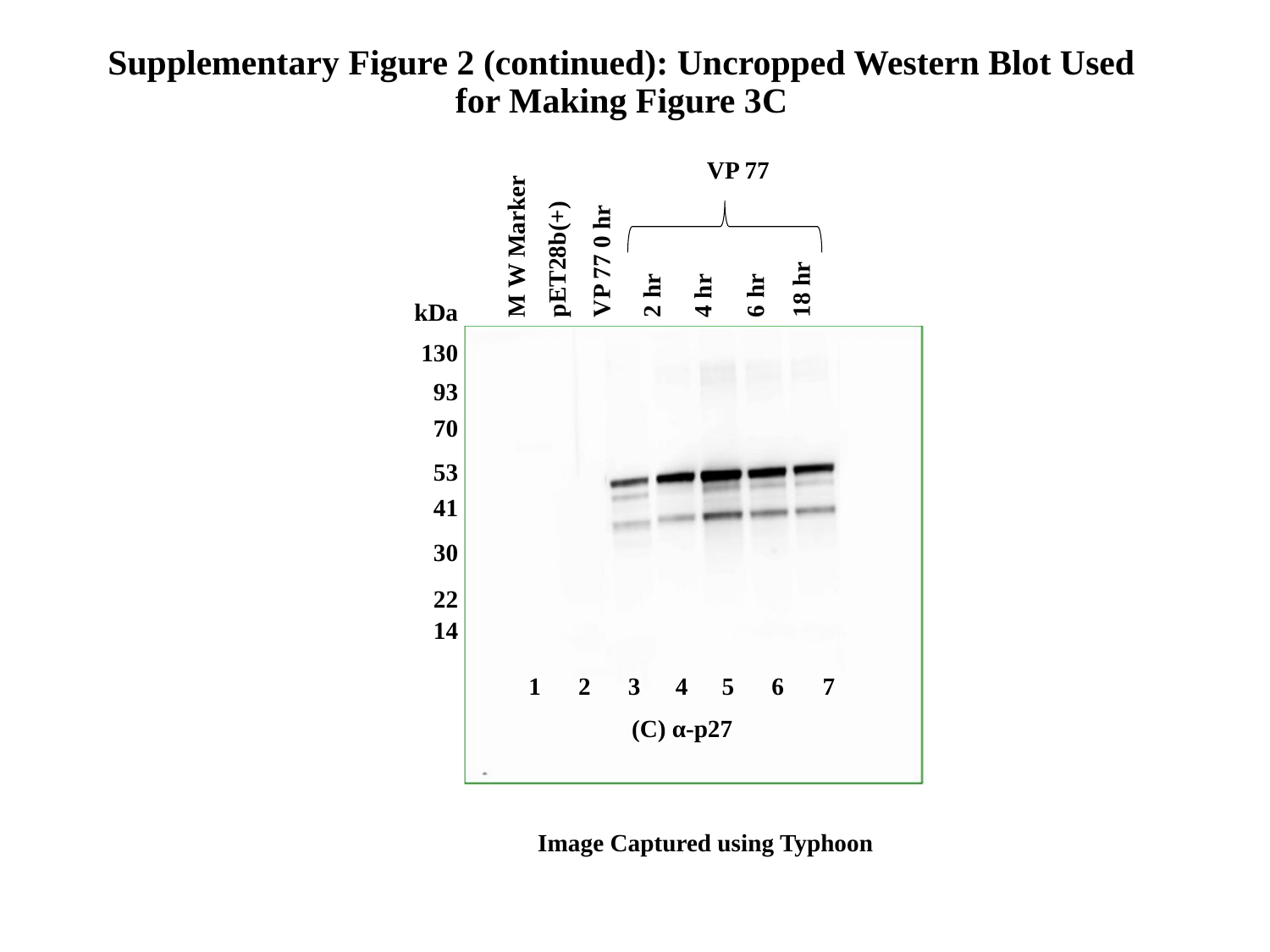

Supplementary Figure 2 (continued): Uncropped Western Blot Used for Making Figure 3C
VP 77
M W Marker
VP 77 0 hr
pET28b(+)
2 hr
4 hr
6 hr
18 hr
 kDa
130
93
70
53
41
30
22
14
1
2
3
4
5
6
7
(C) α-p27
Image Captured using Typhoon
