# Supplementary material for: Expression, purification, and functional characterization of soluble recombinant full-length simian immunodeficiency virus (SIV) Pr55Gag
Source: Heliyon. 2023 Jan 10;9(1):e12892. doi: 10.1016/j.heliyon.2023.e12892 (PMC9853374; doi:10.1016/j.heliyon.2023.e12892)
Supplement: Multimedia component 3 [file mmc3.pptx]

## Slide 1
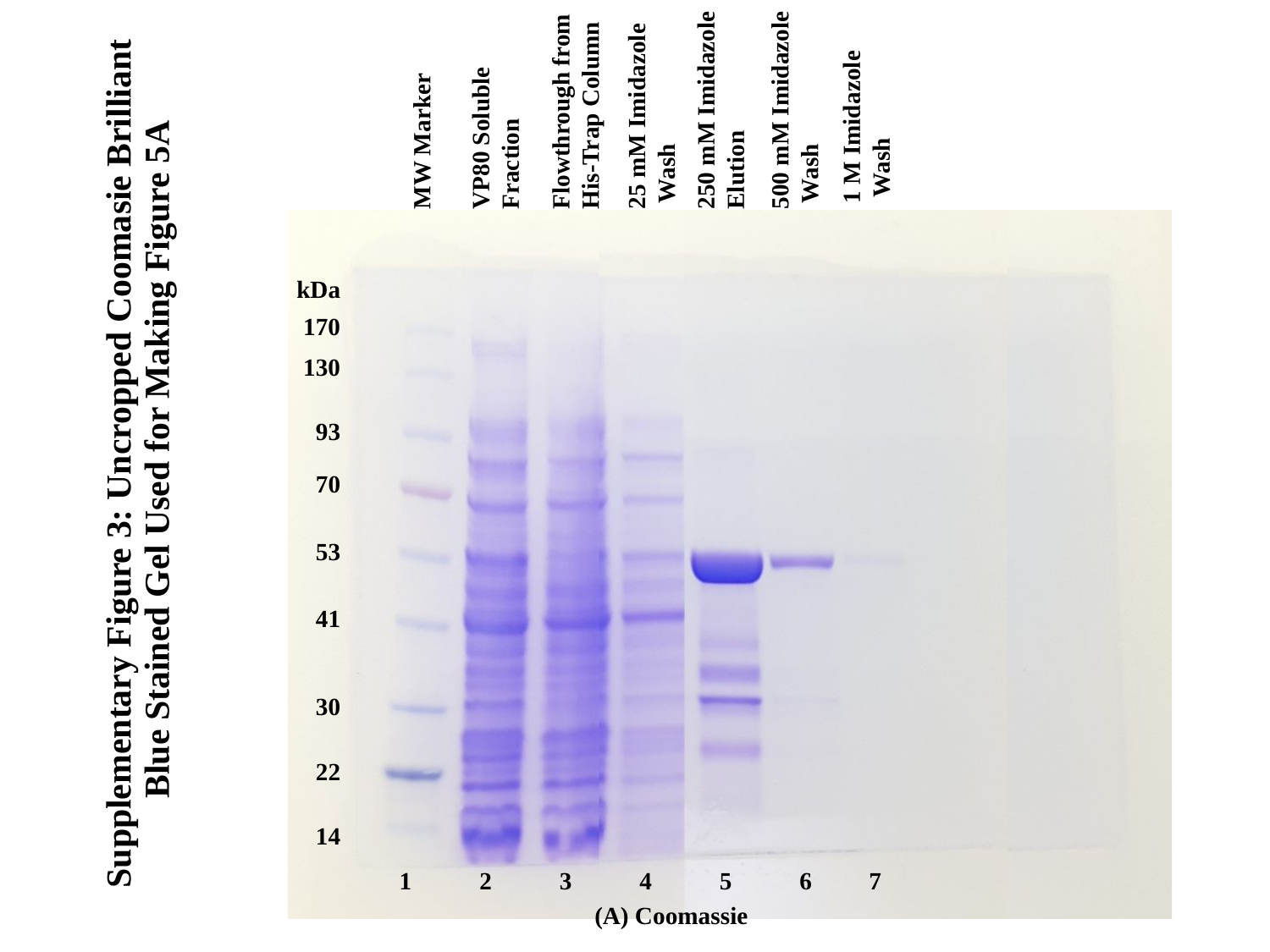

1 M Imidazole
 Wash
500 mM Imidazole
 Wash
250 mM Imidazole Elution
25 mM Imidazole
 Wash
Flowthrough from
His-Trap Column
VP80 Soluble Fraction
MW Marker
 kDa
170
130
93
70
53
41
30
22
14
# Supplementary Figure 3: Uncropped Coomasie Brilliant Blue Stained Gel Used for Making Figure 5A
1
2
3
4
5
6
7
(A) Coomassie

## Slide 2
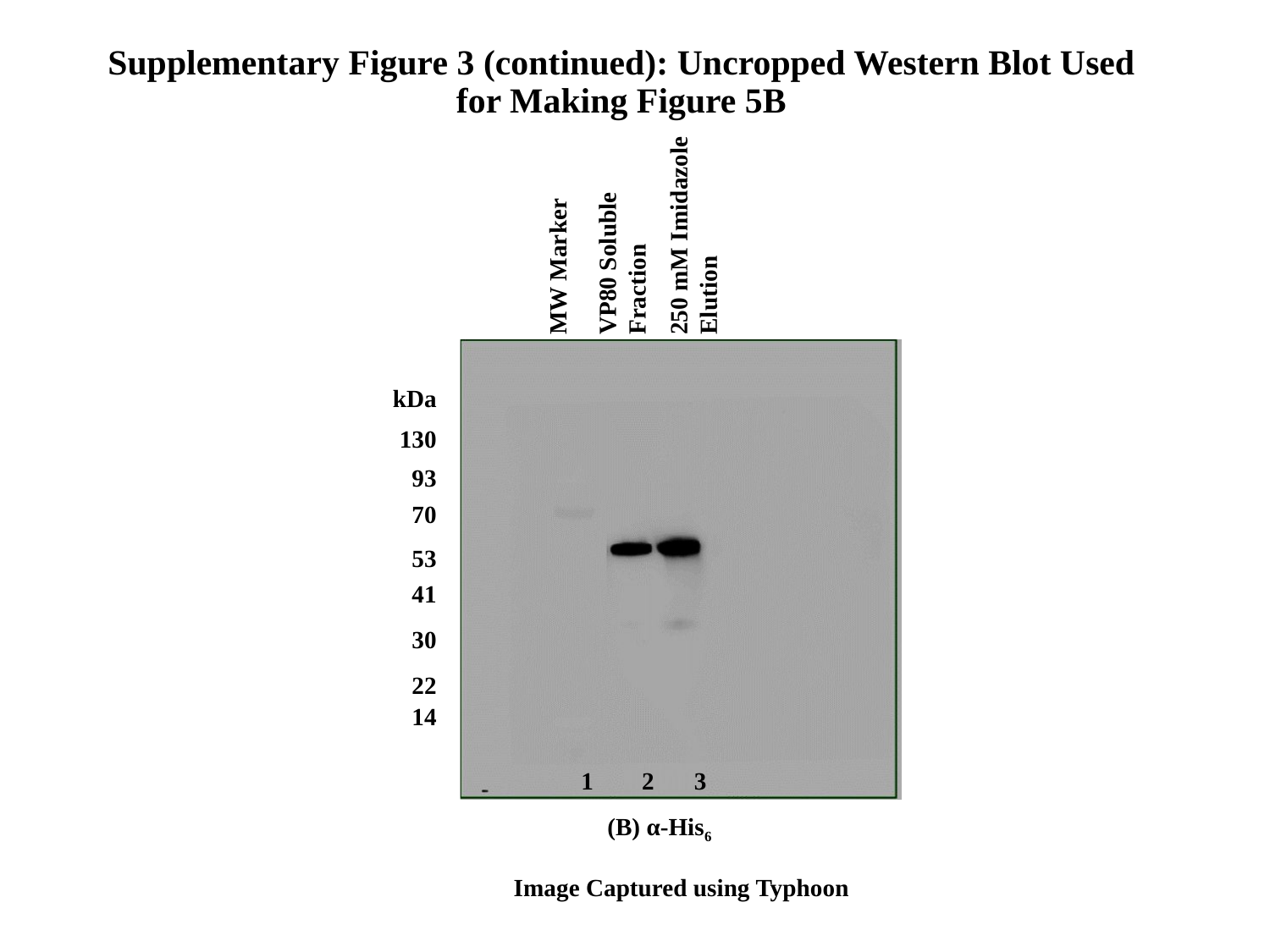

Supplementary Figure 3 (continued): Uncropped Western Blot Used for Making Figure 5B
250 mM Imidazole
Elution
VP80 Soluble Fraction
MW Marker
 kDa
130
93
70
53
41
30
22
14
1
2
3
(B) α-His6
Image Captured using Typhoon

## Slide 3
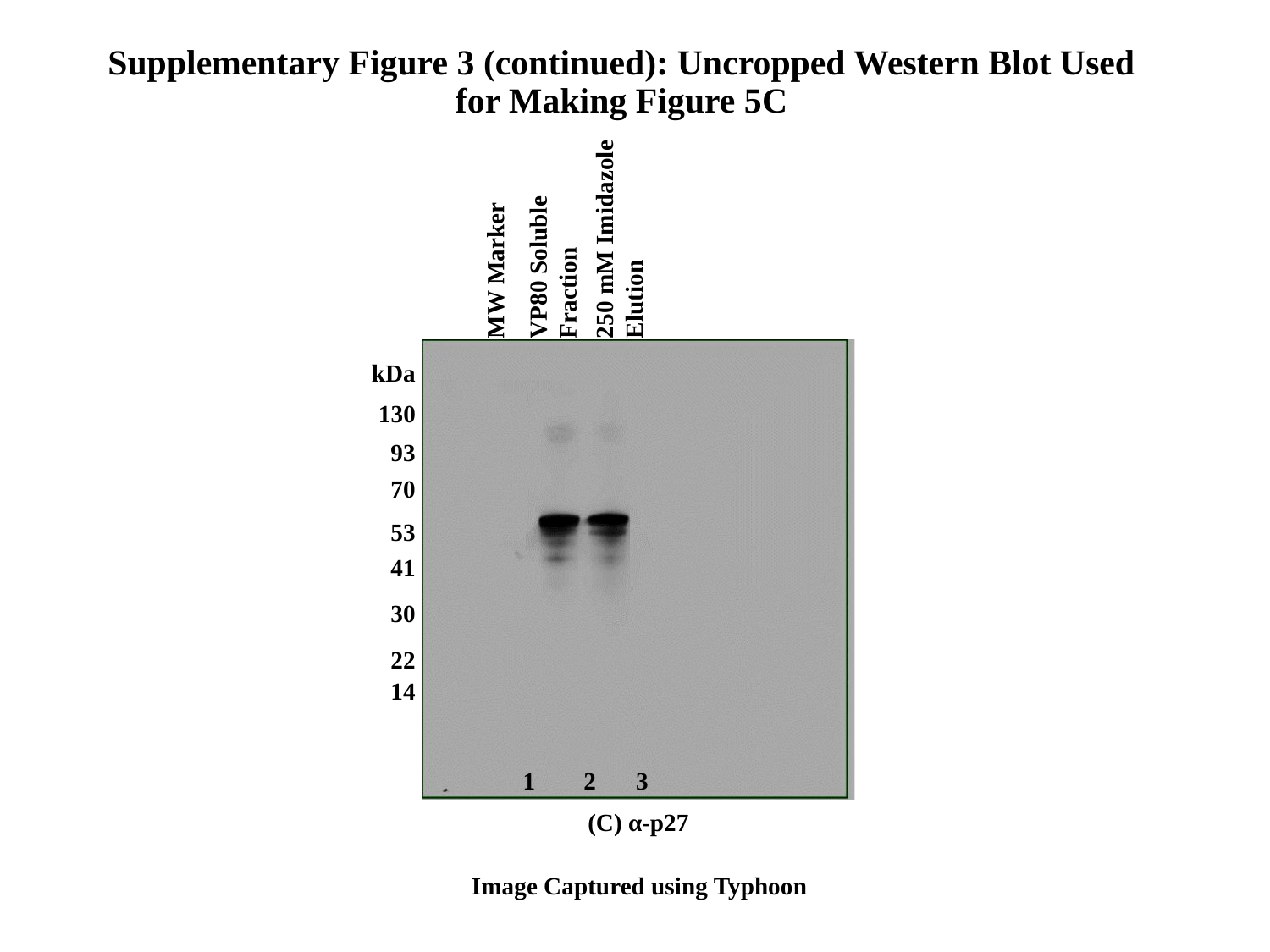

Supplementary Figure 3 (continued): Uncropped Western Blot Used for Making Figure 5C
250 mM Imidazole
Elution
VP80 Soluble Fraction
MW Marker
 kDa
130
93
70
53
41
30
22
14
1
2
3
(C) α-p27
Image Captured using Typhoon
