# Supplementary material for: Expression, purification, and functional characterization of soluble recombinant full-length simian immunodeficiency virus (SIV) Pr55Gag
Source: Heliyon. 2023 Jan 10;9(1):e12892. doi: 10.1016/j.heliyon.2023.e12892 (PMC9853374; doi:10.1016/j.heliyon.2023.e12892)
Supplement: Multimedia component 4 [file mmc4.pptx]

## Slide 1
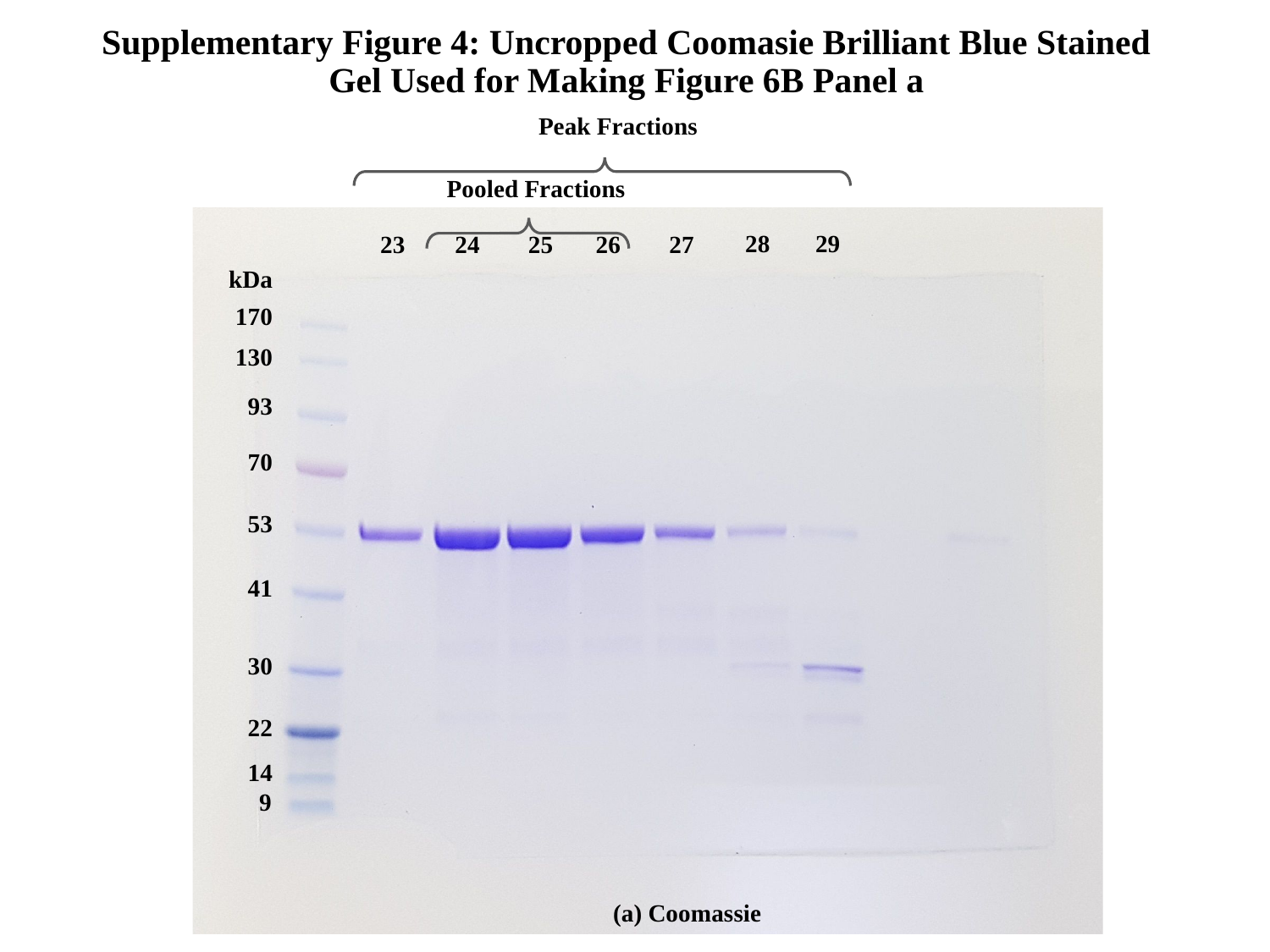

# Supplementary Figure 4: Uncropped Coomasie Brilliant Blue Stained Gel Used for Making Figure 6B Panel a
Peak Fractions
Pooled Fractions
28
29
23
24
25
26
27
 kDa
170
130
93
70
53
41
30
22
14
9
(a) Coomassie

## Slide 2
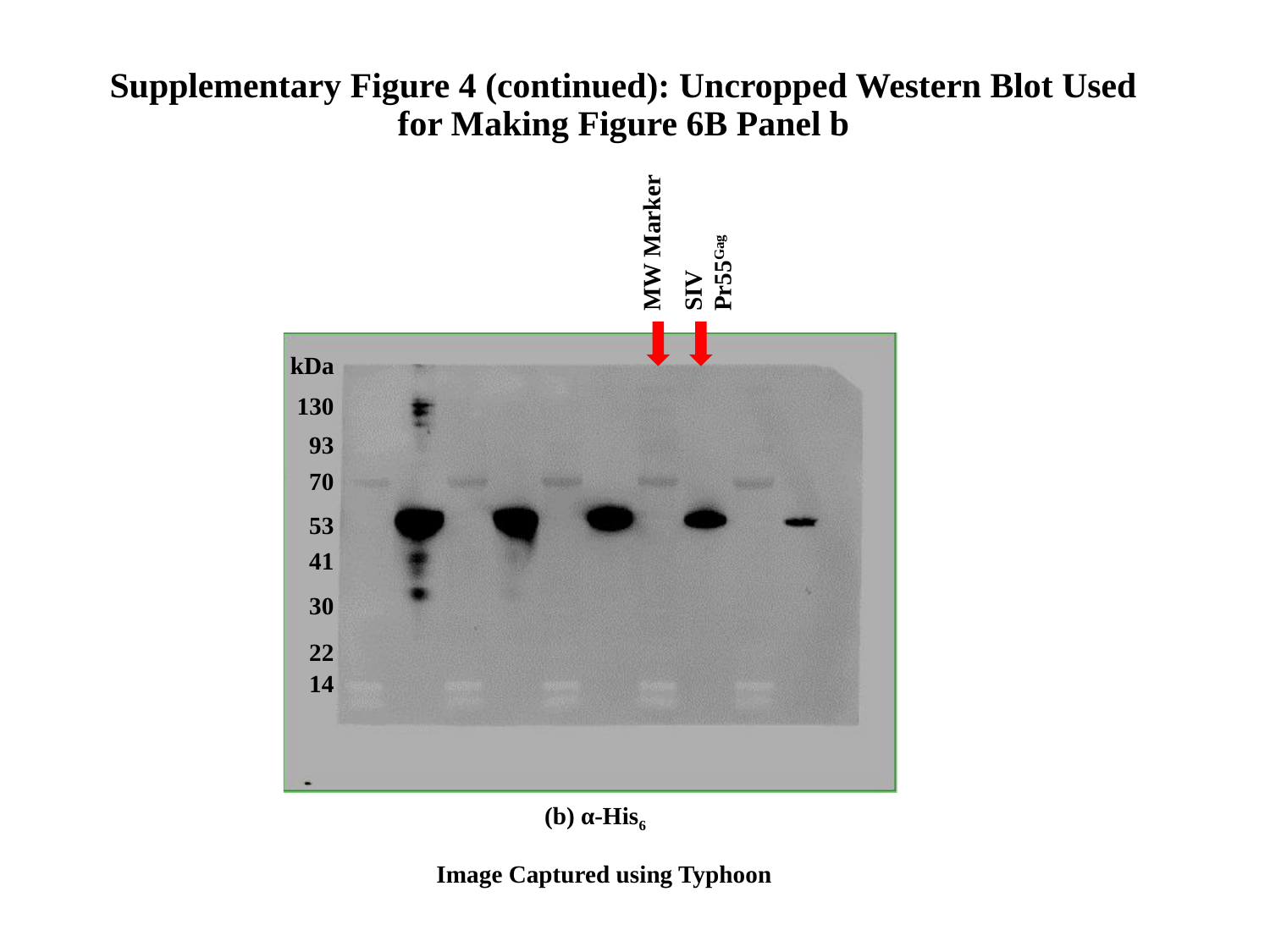

Supplementary Figure 4 (continued): Uncropped Western Blot Used for Making Figure 6B Panel b
MW Marker
SIV Pr55Gag
 kDa
130
93
70
53
41
30
22
14
(b) α-His6
Image Captured using Typhoon

## Slide 3
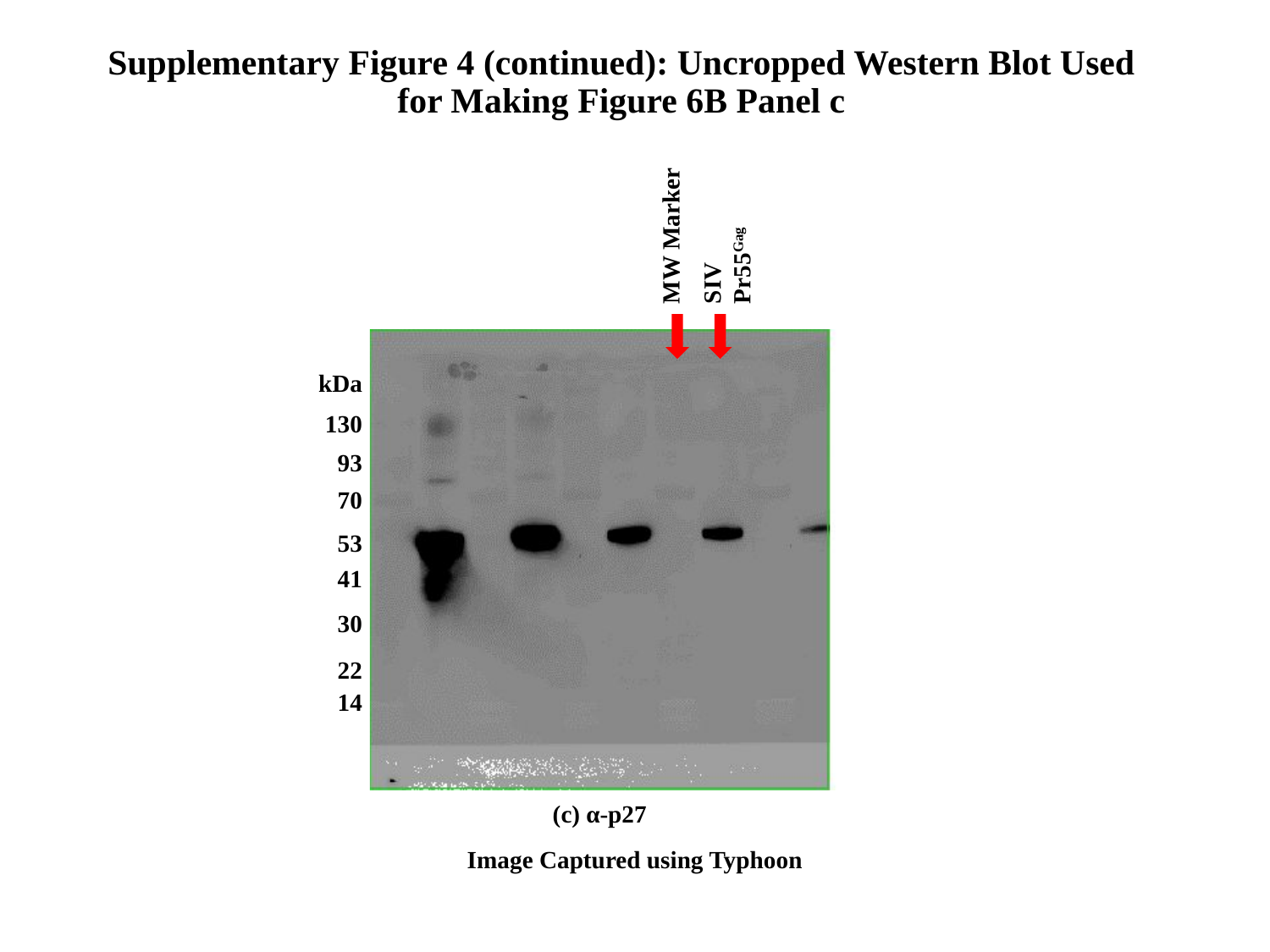

Supplementary Figure 4 (continued): Uncropped Western Blot Used for Making Figure 6B Panel c
MW Marker
SIV Pr55Gag
 kDa
130
93
70
53
41
30
22
14
(c) α-p27
Image Captured using Typhoon
