# Supplementary material for: Expression, purification, and functional characterization of soluble recombinant full-length simian immunodeficiency virus (SIV) Pr55Gag
Source: Heliyon. 2023 Jan 10;9(1):e12892. doi: 10.1016/j.heliyon.2023.e12892 (PMC9853374; doi:10.1016/j.heliyon.2023.e12892)
Supplement: Multimedia component 5 [file mmc5.pptx]

## Slide 1
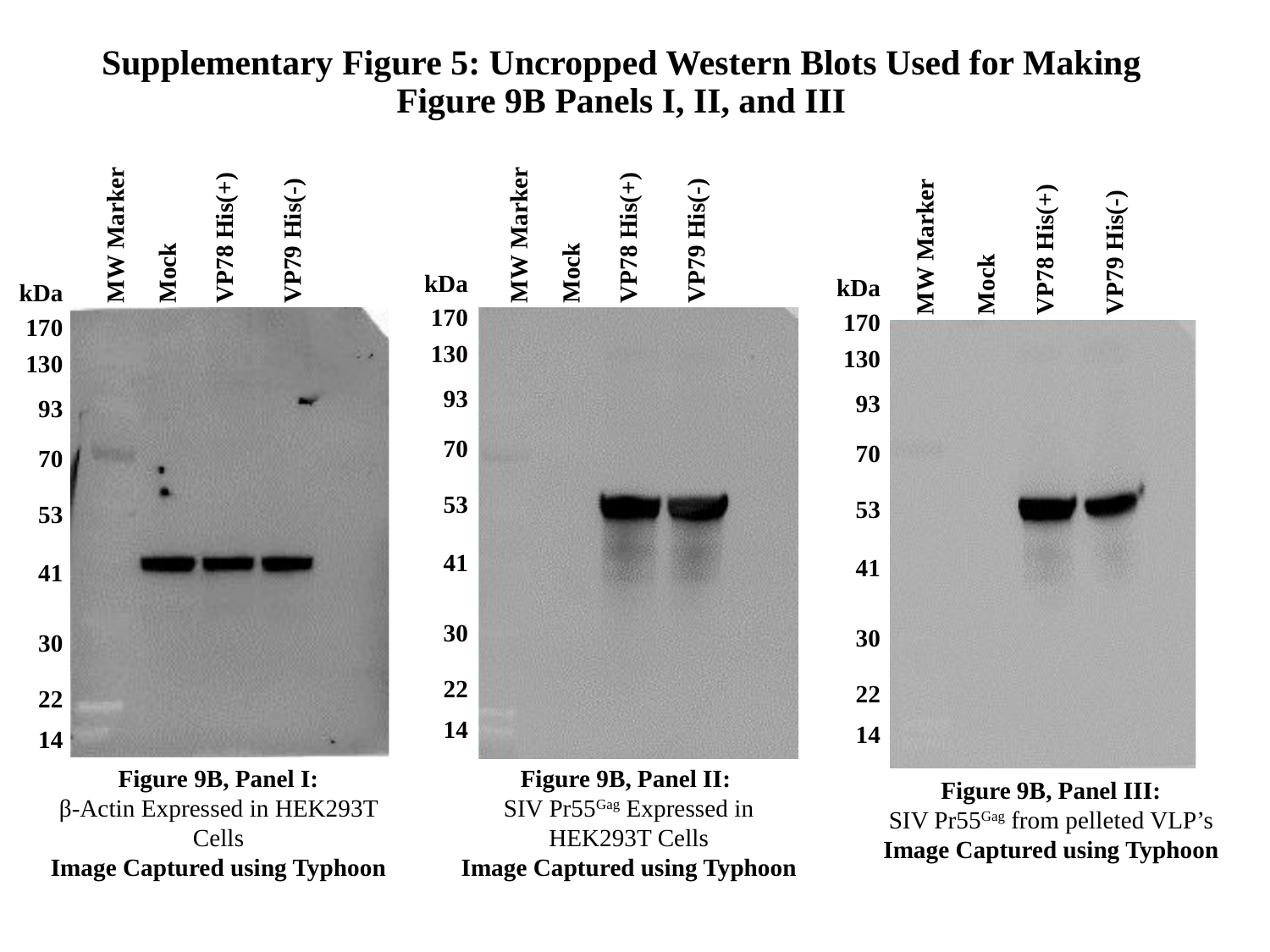

Supplementary Figure 5: Uncropped Western Blots Used for Making Figure 9B Panels I, II, and III
VP78 His(+)
MW Marker
VP79 His(-)
Mock
Figure 9B, Panel II:
SIV Pr55Gag Expressed in HEK293T Cells
Image Captured using Typhoon
VP78 His(+)
MW Marker
VP79 His(-)
Mock
Figure 9B, Panel I:
β-Actin Expressed in HEK293T Cells
Image Captured using Typhoon
VP78 His(+)
MW Marker
VP79 His(-)
Mock
Figure 9B, Panel III:
SIV Pr55Gag from pelleted VLP’s
Image Captured using Typhoon
 kDa
170
130
93
70
53
41
30
22
14
 kDa
170
130
93
70
53
41
30
22
14
 kDa
170
130
93
70
53
41
30
22
14
