# Supplementary material for: Expression, purification, and functional characterization of soluble recombinant full-length simian immunodeficiency virus (SIV) Pr55Gag
Source: Heliyon. 2023 Jan 10;9(1):e12892. doi: 10.1016/j.heliyon.2023.e12892 (PMC9853374; doi:10.1016/j.heliyon.2023.e12892)
Supplement: Multimedia component 6 [file mmc6.docx]

| **Supplementary Table 1: Description of the primers used for cloning, sequencing, conventional PCR and real time PCR.** | | | | | | |
| --- | --- | --- | --- | --- | --- | --- |
| **Clone Name** | **Primer Name** | **Description of the primer** | ***S or AS** | **Primer Sequence** | **Nucleotide position** | **Template Plasmid** |
| VP78 | OTR 1549 | SIV Gag with Kozak and His_6_-tag | S | 5’ ccg **CTC GAG** GCC GCC ACC *ATG* GGC GTG AGA AAC TCC GTC 3’ | SIVmac239  1309 - 1329 | VP77 |
|  | OTR 1550 |  | AS | 5’ T TCT CTC TTT GGA GGA GAC CAG CAC CAC CAC CAC CAC CAC *TAG* **CTC GAG** cgg 3’ | SIVmac239  2817-2838 |  |
| VP79 | OTR 1549 | SIV Gag with Kozak and without His_6_-tag | S | 5’ ccg **CTC GAG** GCC GCC ACC *ATG* GGC GTG AGA AAC TCC GTC 3’ | SIVmac239  1309 - 1329 | VP77 |
|  | OTR1551 |  | AS | 5’ T CTC TTT GGA GGA GAC CAG *TAG* **CTC GAG** cgg 3’ | SIVmac239  2820-2841 |  |
| OTR 1549 | | Sequencing primer | S | 5’ CCG CTC GAG GCC GCC ACC ATG GGC GTG AGA AAC TCC GTC 3’ | SIVmac239  1309 - 1329 | |
| OTR 1552 | | Sequencing primer | AS | 5’ CTT TTC CTC TAT CAA TTT TAC 3’ | SIVmac239  1780 - 1800 | |
| OTR 1553 | | Sequencing primer | S | 5’ GTC AGG ATC AGA TAT TGC AGG 3’ | SIVmac239  2001 - 2021 | |
| OTR 1554 | | Sequencing primer | S | 5’ AAG AGG GAC ACT CTG CAA GG 3’ | SIVmac239  2501 - 2520 | |

**Sequence in lowercase** - Dummy sequence for the restriction enzyme.

**Sequence in bold -** Restriction enzyme sequence present in the oligos that were employed for cloning.

**Sequence in underlined -** Kozak sequence/Hexa-Histidine tag

**Sequence in italics** - Start codon/Stop codon

| **Primers used for RT-qPCR** | | | | |
| --- | --- | --- | --- | --- |
| **Primer** | **Description** | ***S or AS** | **Sequence** | **Nucleotide position or reference** |
| OTR 1650 | Vector specific  and  SYBR-Green qPCR Forward primer | S | 5’ CCC TTT CTG CTT TGG GAA ACC G 3’ | SIVmac239  1033 - 1054 |
| OTR 1651 | Vector specific  and  SYBR-Green qPCR Reverse primer | AS | 5’ CCC ACT CTA TCT TAT TAC CC 3’ | SIVmac239  1287 - 1306 |
| OFM 456 | β-actin  SYBR-Green qPCR  Forward primer | S | 5’ GGC GGC ACC ACC ATG TAC CCT 3’ | Human β-actin  (GenBank Accession Number NM_001101.3)  985 - 1005 |
| OTR 1199 | β-actin  SYBR-Green qPCR  Reverse primer | AS | 5’ TGC TCT AGA CTC GAG CTA GAA GCA TTT GCG GTG G 3’ | Human β-actin  (GenBank Accession Number NM_001101.3)  985 - 1005 |

*****S - Sense; AS - Antisense.
