# Supplementary material for: Expression, purification, and functional characterization of soluble recombinant full-length simian immunodeficiency virus (SIV) Pr55Gag
Source: Heliyon. 2023 Jan 10;9(1):e12892. doi: 10.1016/j.heliyon.2023.e12892 (PMC9853374; doi:10.1016/j.heliyon.2023.e12892)
Supplement: Multimedia component 7 [file mmc7.docx]

**Supplementary Table 2: Sequence of SIV Pr55^Gag^ cloned into the expression plasmids.**

| **Plasmid** | **Description** | **Sequence** | **Landmarks** |
| --- | --- | --- | --- |
| **VP77** | SIV Pr55^Gag^ in pET28b(+) with His_6_-tag | **CCATGG**GCGTGAGAAACTCCGTCTTGTCAGGGAAGAAAGCAGATGAATTAGAAAAAATTAGGCTACGACCCAACGGAAAGAAAAAGTACATGTTGAAGCATGTAGTATGGGCAGCAAATGAATTAGATAGATTTGGATTAGCAGAAAGCCTGTTGGAGAACAAAGAAGGATGTCAAAAAATACTTTCGGTCTTAGCTCCATTAGTGCCAACAGGCTCAGAAAATTTAAAAAGCCTTTATAATACTGTCTGCGTCATCTGGTGCATTCACGCAGAAGAGAAAGTGAAACACACTGAGGAAGCAAAACAGATAGTGCAGAGACACCTAGTGGTGGAAACAGGAACAACAGAAACTATGCCAAAAACAAGTAGACCAACAGCACCATCTAGCGGCAGAGGAGGAAATTACCCAGTACAACAAATAGGTGGTAACTATGTCCACCTGCCATTAAGCCCGAGAACATTAAATGCCTGGGTAAAATTGATAGAGGAAAAGAAATTTGGAGCAGAAGTAGTGCCAGGATTTCAGGCACTGTCAGAAGGTTGCACCCCCTATGACATTAATCAGATGTTAAATTGTGTGGGAGACCATCAAGCGGCTATGCAGATTATCAGAGATATTATAAACGAGGAGGCTGCAGATTGGGACTTGCAGCACCCACAACCAGCTCCACAACAAGGACAACTTAGGGAGCCGTCAGGATCAGATATTGCAGGAACAACTAGTTCAGTAGATGAACAAATCCAGTGGATGTACAGACAACAGAACCCCATACCAGTAGGCAACATTTACAGGAGATGGATCCAACTGGGGTTGCAAAAATGTGTCAGAATGTATAACCCAACAAACATTCTAGATGTAAAACAAGGGCCAAAAGAGCCATTTCAGAGCTATGTAGACAGGTTCTACAAAAGTTTAAGAGCAGAACAGACAGATGCAGCAGTAAAGAATTGGATGACTCAAACACTGCTGATTCAAAATGCTAACCCAGATTGCAAGCTAGTGCTGAAGGGGCTGGGTGTGAATCCCACCCTAGAAGAAATGCTGACGGCTTGTCAAGGAGTAGGGGGGCCGGGACAGAAGGCTAGATTAATGGCAGAAGCCCTGAAAGAGGCCCTCGCACCAGTGCCAATCCCTTTTGCAGCAGCCCAACAGAGGGGACCAAGAAAGCCAATTAAGTGTTGGAATTGTGGGAAAGAGGGACACTCTGCAAGGCAATGCAGAGCCCCAAGAAGACAGGGATGCTGGAAATGTGGAAAAATGGACCATGTTATGGCCAAATGCCCAGACAGACAGGCGGGTTTTTTAGGCCTTGGTCC**G**TGGGGAAAGAAGCCCCGCAATTTCCCA**A**TGGCTCAAGTGCATCAGGGGCTGATGCCAACTGCTCCCCCAGAGGACCCAGCTGTGGATCTGCTAAAGAACTACATGCAGTTGGGCAAGCAGCAGAGAGAAAAGCAGAGAGAAAGCAGAGAGAAGCCTTACAAGGAGGTGACAGAGGATTTGCTGCACCTCAATTCTCTCTTTGGAGGAGACCAG**CTCGAG*CACCACCACCACCACCAC*TAG** | - **CCATGG -***Nco*I site in pET28b(+) used for cloning - **ATG -** Gag start codon - CC**G**TGG and CA**A**TGG - Silently mutated to inactivate inherent *NCo*I sites without changing the amino acid sequence - **CTCGAG -** *Xho*I site in pET28b(+) used for cloning - ***CACCACCACCACCACCAC*** - His_6_-tag - **TAG** - Gag stop codon |
| **VP78** | SIV Pr55^Gag^ in pcDNA3 with His_6_-tag | **CTCGAG*GCCGCCACC*ATG**GGCGTGAGAAACTCCGTCTTGTCAGGGAAGAAAGCAGATGAATTAGAAAAAATTAGGCTACGACCCAACGGAAAGAAAAAGTACATGTTGAAGCATGTAGTATGGGCAGCAAATGAATTAGATAGATTTGGATTAGCAGAAAGCCTGTTGGAGAACAAAGAAGGATGTCAAAAAATACTTTCGGTCTTAGCTCCATTAGTGCCAACAGGCTCAGAAAATTTAAAAAGCCTTTATAATACTGTCTGCGTCATCTGGTGCATTCACGCAGAAGAGAAAGTGAAACACACTGAGGAAGCAAAACAGATAGTGCAGAGACACCTAGTGGTGGAAACAGGAACAACAGAAACTATGCCAAAAACAAGTAGACCAACAGCACCATCTAGCGGCAGAGGAGGAAATTACCCAGTACAACAAATAGGTGGTAACTATGTCCACCTGCCATTAAGCCCGAGAACATTAAATGCCTGGGTAAAATTGATAGAGGAAAAGAAATTTGGAGCAGAAGTAGTGCCAGGATTTCAGGCACTGTCAGAAGGTTGCACCCCCTATGACATTAATCAGATGTTAAATTGTGTGGGAGACCATCAAGCGGCTATGCAGATTATCAGAGATATTATAAACGAGGAGGCTGCAGATTGGGACTTGCAGCACCCACAACCAGCTCCACAACAAGGACAACTTAGGGAGCCGTCAGGATCAGATATTGCAGGAACAACTAGTTCAGTAGATGAACAAATCCAGTGGATGTACAGACAACAGAACCCCATACCAGTAGGCAACATTTACAGGAGATGGATCCAACTGGGGTTGCAAAAATGTGTCAGAATGTATAACCCAACAAACATTCTAGATGTAAAACAAGGGCCAAAAGAGCCATTTCAGAGCTATGTAGACAGGTTCTACAAAAGTTTAAGAGCAGAACAGACAGATGCAGCAGTAAAGAATTGGATGACTCAAACACTGCTGATTCAAAATGCTAACCCAGATTGCAAGCTAGTGCTGAAGGGGCTGGGTGTGAATCCCACCCTAGAAGAAATGCTGACGGCTTGTCAAGGAGTAGGGGGGCCGGGACAGAAGGCTAGATTAATGGCAGAAGCCCTGAAAGAGGCCCTCGCACCAGTGCCAATCCCTTTTGCAGCAGCCCAACAGAGGGGACCAAGAAAGCCAATTAAGTGTTGGAATTGTGGGAAAGAGGGACACTCTGCAAGGCAATGCAGAGCCCCAAGAAGACAGGGATGCTGGAAATGTGGAAAAATGGACCATGTTATGGCCAAATGCCCAGACAGACAGGCGGGTTTTTTAGGCCTTGGTCC**G**TGGGGAAAGAAGCCCCGCAATTTCCCA**A**TGGCTCAAGTGCATCAGGGGCTGATGCCAACTGCTCCCCCAGAGGACCCAGCTGTGGATCTGCTAAAGAACTACATGCAGTTGGGCAAGCAGCAGAGAGAAAAGCAGAGAGAAAGCAGAGAGAAGCCTTACAAGGAGGTGACAGAGGATTTGCTGCACCTCAATTCTCTCTTTGGAGGAGACCAG***CACCACCACCACCACCAC*TAGCTCGAG** | - **CTCGAG -***Xho*I sites in pcDNA3 used for cloning - *GCCGCCACC* – Kozak sequence - **ATG -** Gag start codon - CC**G**TGG and CA**A**TGG - Silently mutated to inactivate inherent *NCo*I sites without changing the amino acid sequence - ***CACCACCACCACCACCAC*** - His_6_-tag - **TAG** - Gag stop codon |
| **VP79** | SIV Pr55^Gag^ in pcDNA3 without His_6_-tag | **CTCGAG*GCCGCCACC*ATG**GGCGTGAGAAACTCCGTCTTGTCAGGGAAGAAAGCAGATGAATTAGAAAAAATTAGGCTACGACCCAACGGAAAGAAAAAGTACATGTTGAAGCATGTAGTATGGGCAGCAAATGAATTAGATAGATTTGGATTAGCAGAAAGCCTGTTGGAGAACAAAGAAGGATGTCAAAAAATACTTTCGGTCTTAGCTCCATTAGTGCCAACAGGCTCAGAAAATTTAAAAAGCCTTTATAATACTGTCTGCGTCATCTGGTGCATTCACGCAGAAGAGAAAGTGAAACACACTGAGGAAGCAAAACAGATAGTGCAGAGACACCTAGTGGTGGAAACAGGAACAACAGAAACTATGCCAAAAACAAGTAGACCAACAGCACCATCTAGCGGCAGAGGAGGAAATTACCCAGTACAACAAATAGGTGGTAACTATGTCCACCTGCCATTAAGCCCGAGAACATTAAATGCCTGGGTAAAATTGATAGAGGAAAAGAAATTTGGAGCAGAAGTAGTGCCAGGATTTCAGGCACTGTCAGAAGGTTGCACCCCCTATGACATTAATCAGATGTTAAATTGTGTGGGAGACCATCAAGCGGCTATGCAGATTATCAGAGATATTATAAACGAGGAGGCTGCAGATTGGGACTTGCAGCACCCACAACCAGCTCCACAACAAGGACAACTTAGGGAGCCGTCAGGATCAGATATTGCAGGAACAACTAGTTCAGTAGATGAACAAATCCAGTGGATGTACAGACAACAGAACCCCATACCAGTAGGCAACATTTACAGGAGATGGATCCAACTGGGGTTGCAAAAATGTGTCAGAATGTATAACCCAACAAACATTCTAGATGTAAAACAAGGGCCAAAAGAGCCATTTCAGAGCTATGTAGACAGGTTCTACAAAAGTTTAAGAGCAGAACAGACAGATGCAGCAGTAAAGAATTGGATGACTCAAACACTGCTGATTCAAAATGCTAACCCAGATTGCAAGCTAGTGCTGAAGGGGCTGGGTGTGAATCCCACCCTAGAAGAAATGCTGACGGCTTGTCAAGGAGTAGGGGGGCCGGGACAGAAGGCTAGATTAATGGCAGAAGCCCTGAAAGAGGCCCTCGCACCAGTGCCAATCCCTTTTGCAGCAGCCCAACAGAGGGGACCAAGAAAGCCAATTAAGTGTTGGAATTGTGGGAAAGAGGGACACTCTGCAAGGCAATGCAGAGCCCCAAGAAGACAGGGATGCTGGAAATGTGGAAAAATGGACCATGTTATGGCCAAATGCCCAGACAGACAGGCGGGTTTTTTAGGCCTTGGTCC**G**TGGGGAAAGAAGCCCCGCAATTTCCCA**A**TGGCTCAAGTGCATCAGGGGCTGATGCCAACTGCTCCCCCAGAGGACCCAGCTGTGGATCTGCTAAAGAACTACATGCAGTTGGGCAAGCAGCAGAGAGAAAAGCAGAGAGAAAGCAGAGAGAAGCCTTACAAGGAGGTGACAGAGGATTTGCTGCACCTCAATTCTCTCTTTGGAGGAGACCAG**TAGCTCGAG** | - **CTCGAG -***Xho*I sites in pcDNA3 used for cloning - *GCCGCCACC* – Kozak sequence - **ATG -** Gag start codon - CC**G**TGG and CA**A**TGG - Silently mutated to inactivate inherent *NCo*I sites without changing the amino acid sequence **TAG** - Gag stop codon |
| **VP80** | SIV Pr55^Gag^ with RBS site silently mutated in pET28b(+) with His_6_-tag | **CCATGG**GCGTGAGAAACTCCGTCTTGTCAGGGAAGAAAGCAGATGAATTAGAAAAAATTAGGCTACGACCCAACGGAAAGAAAAAGTACATGTTGAAGCATGTAGTATGGGCAGCAAATGAATTAGATAGATTTGGATTAGCAGAAAGCCTGTTGGAGAACAAAGAAGGATGTCAAAAAATACTTTCGGTCTTAGCTCCATTAGTGCCAACAGGCTCAGAAAATTTAAAAAGCCTTTATAATACTGTCTGCGTCATCTGGTGCATTCACGCAGAAGAGAAAGTGAAACACACTGAGGAAGCAAAACAGATAGTGCAGAGACACCTAGTGGTGGAAAC**C**GG**C**AC**T**AC**C**GAAACT**ATG**CCAAAAACAAGTAGACCAACAGCACCATCTAGCGGCAGAGGAGGAAATTACCCAGTACAACAAATAGGTGGTAACTATGTCCACCTGCCATTAAGCCCGAGAACATTAAATGCCTGGGTAAAATTGATAGAGGAAAAGAAATTTGGAGCAGAAGTAGTGCCAGGATTTCAGGCACTGTCAGAAGGTTGCACCCCCTATGACATTAATCAGATGTTAAATTGTGTGGGAGACCATCAAGCGGCTATGCAGATTATCAGAGATATTATAAACGAGGAGGCTGCAGATTGGGACTTGCAGCACCCACAACCAGCTCCACAACAAGGACAACTTAGGGAGCCGTCAGGATCAGATATTGCAGGAACAACTAGTTCAGTAGATGAACAAATCCAGTGGATGTACAGACAACAGAACCCCATACCAGTAGGCAACATTTACAGGAGATGGATCCAACTGGGGTTGCAAAAATGTGTCAGAATGTATAACCCAACAAACATTCTAGATGTAAAACAAGGGCCAAAAGAGCCATTTCAGAGCTATGTAGACAGGTTCTACAAAAGTTTAAGAGCAGAACAGACAGATGCAGCAGTAAAGAATTGGATGACTCAAACACTGCTGATTCAAAATGCTAACCCAGATTGCAAGCTAGTGCTGAAGGGGCTGGGTGTGAATCCCACCCTAGAAGAAATGCTGACGGCTTGTCAAGGAGTAGGGGGGCCGGGACAGAAGGCTAGATTAATGGCAGAAGCCCTGAAAGAGGCCCTCGCACCAGTGCCAATCCCTTTTGCAGCAGCCCAACAGAGGGGACCAAGAAAGCCAATTAAGTGTTGGAATTGTGGGAAAGAGGGACACTCTGCAAGGCAATGCAGAGCCCCAAGAAGACAGGGATGCTGGAAATGTGGAAAAATGGACCATGTTATGGCCAAATGCCCAGACAGACAGGCGGGTTTTTTAGGCCTTGGTCC**G**TGGGGAAAGAAGCCCCGCAATTTCCCA**A**TGGCTCAAGTGCATCAGGGGCTGATGCCAACTGCTCCCCCAGAGGACCCAGCTGTGGATCTGCTAAAGAACTACATGCAGTTGGGCAAGCAGCAGAGAGAAAAGCAGAGAGAAAGCAGAGAGAAGCCTTACAAGGAGGTGACAGAGGATTTGCTGCACCTCAATTCTCTCTTTGGAGGAGACCAG**CTCGAG*CACCACCACCACCACCAC*TAG** | - **CCATGG -***NcoI* site in pET28b(+) used for cloning - **ATG** - Gag start codon - AC**C**GG**C**AC**T**AC**C -** Silently mutated RBS site - **ATG -** Second in-frame start codon - CC**G**TGG and CA**A**TGG - Silently mutated to inactivate inherent *NCo*I sites without changing the amino acid sequence - **CTCGAG -** *Xho*I site in pET28b(+) used for cloning - ***CACCACCACCACCACCAC*** - His_6_-tag - TAG - Gag stop codon |
